# Long‐Term Demographic Trends of Near Threatened Coastal Dolphins Living in an Urban Estuary

**DOI:** 10.1002/ece3.70834

**Published:** 2025-01-06

**Authors:** Kennadie Haigh, Guido J. Parra, Luciana Möller, Aude Steiner, Mike Bossley

**Affiliations:** ^1^ Cetacean Ecology, Behaviour and Evolution Lab, College of Science and Engineering Flinders University Adelaide South Australia Australia; ^2^ Bressaucourt Switzerland; ^3^ Whale and Dolphin Conservation Adelaide South Australia Australia

**Keywords:** coastal dolphins, mark‐recapture, population demography, residence, site fidelity, *Tursiops aduncus*

## Abstract

Understanding population demography of threatened species and how they vary in relation to natural and anthropogenic stressors is essential for effective conservation. We used a long‐term photographic capture‐recapture dataset (1993–2020) of Indo‐Pacific bottlenose dolphins ( *Tursiops aduncus* ) in the highly urbanised Adelaide Dolphin Sanctuary (ADS), South Australia, to estimate key demographic parameters and their variability over time. These parameters were analysed in relation to environmental variables used as indicators of local and large‐scale climatic events. Our findings indicate that apparent survival was high (0.98–0.99) and did not vary seasonally. Estimates of abundance were not directly related to environmental variables but were linked to seasonal temporary emigration. Abundance peaked in summer with an average of 85.37 dolphins (SD = 30.23) and was lowest in winter, with 68.57 (SD = 24.70) individuals. Site fidelity at the population level was low, but lagged identification rates revealed a population of approximately 28 individuals at any one time. Trend analysis suggests an increase in dolphin abundance from 1993 and persistence of the population over decades despite significant urbanisation, although numbers have declined in more recent years. Further research is needed to understand the cumulative impacts leading to this population decline and to assess its future viability under different management scenarios. Conservation strategies aimed at increasing reproductive rates and promoting connectivity to adjacent waters are likely to be more effective in reversing population declines.

## Graphical abstract

A long‐term photographic capture‐recapture dataset (1993–2020) of Indo‐Pacific bottlenose dolphins ( *Tursiops aduncus* ) in a highly urbanised habitat was used to estimate key demographic parameters and their variability over time. Trend analysis shows long‐term population persistence with seasonal fluctuations in abundance driven by temporary emigration movements however, cumulative impacts may be driving a recent decline in dolphin abundance. Improving reproductive rates and connectivity may help reverse population decline.

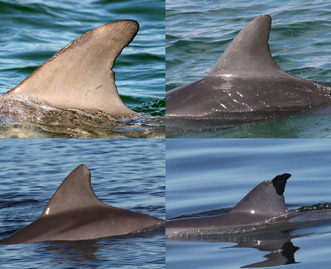

## 1 Introduction

Coastlines are popular locations for residential, economic, and industrial activities (Alter et al. 2021; Todd et al. 2019). Urbanisation can affect population demographics of species living among these areas, either directly or indirectly, and over varying timescales through resource extraction, pollution, and habitat modification (Magera et al. 2013; Mayer‐Pinto et al. 2018). Large‐scale global climate factors add to the cumulative impacts affecting coastal ecosystems (He and Silliman 2019), and as a result, coastal ecosystems are among the most threatened ecosystems in the world (Alter et al. 2021).

Numerous dolphin species inhabit coastal and estuarine ecosystems (Braulik et al. 2023); however, a general understanding of demographic processes and how they vary in relation to natural and anthropogenic disturbances remains limited (Pirotta et al. 2023). Understanding the impacts of human activities on long‐lived species like dolphins requires extensive, long‐term monitoring, which is uncommon in marine mammal studies (Cheney et al. 2014; Cagnazzi et al. 2020). These top‐ and meso‐level predators can serve as indicators of ecosystem health, with their population dynamics reflecting the underlying ecosystem health (Wells et al. 2004). As such, demographic information of marine predators may assist effective marine ecosystem‐based management (Pirotta et al. 2023).

Indo‐Pacific bottlenose dolphins ( *Tursiops aduncus* ) (hereafter bottlenose dolphins) are found in coastal waters throughout the Indian and western Pacific Oceans (Wang and Yang 2009; Braulik et al. 2019). Due to their life history traits (i.e., long‐lived, late maturity, and slow breeders), frequent overlap with areas of intense anthropogenic activities, and generally small/localised populations, this species is currently listed as ‘Near Threatened’ on the IUCN Red List (Braulik et al. 2019). In Australia, they can be found along the entire Australian coast, with several populations living near major urban developments (e.g., Fury and Harrison 2008; Ansmann et al. 2013; Palmer et al. 2014; Zanardo, Parra, and Möller 2016). Minimum population estimates across Australia vary depending on the size of the study area but can range from less than 50 individuals in the Swan Canning Riverpark (55 km^2^), Western Australia (Chabanne et al. 2017), to 700–1000 individuals off North Stradbroke Island (150 km^2^), Queensland (Chilvers and Corkeron 2003), and population demographic information is missing for most populations across their Australian range (Bilgmann et al. 2019). In South Australia, two genetic populations have been identified in the two major South Australian gulfs, Spencer Gulf and Gulf St Vincent (Pratt et al. 2018). An anthropogenic threat assessment based on expert elicitation showed that climate change posed the greatest overall threat in Spencer Gulf (and likely the same for Gulf St Vincent) to marine wildlife, including bottlenose dolphins (Robbins et al. 2017). However, no quantitative studies have investigated the influence of local and large‐scale climate variability on the population demographics of resident marine top predators in this region.

Along the metropolitan coastline of South Australia, bottlenose dolphins occur in the Port Adelaide River estuary (Port River hereafter), Barker Inlet, and Gulf St Vincent (Bossley et al. 2017; Zanardo, Parra, and Möller 2016; Bilgmann et al. 2019). The Port River—Barker Inlet estuary is situated approximately 15 km northwest of the Adelaide CBD and is the main manufacturing site and shipping port for the state dating back to 1840, making it a long‐standing, highly urbanised environment with plans for future developments. Dolphins living in this area are subject to anthropogenic threats, including pollution from industrial/agricultural runoff, sewerage effluent, stormwater runoff, heat effluent, boat strike, entanglement in nets and fishing equipment, noise pollution, dredging, and marine debris (Bossley et al. 2017; Kemper et al. 2005). Additionally, the local dolphin population has been subject to deliberate attacks. In response to these threats the Adelaide Dolphin Sanctuary (ADS) was established in the Port River in 2005 with a mandate to protect both the dolphin population and the habitat that sustains them (Bossley et al. 2017; Department for Environment and Heritage 2008). Despite this protection, a comprehensive understanding about ADS dolphin demographics has not been reached (Department for Environment and Heritage 2008).

This study used a long‐term photographic capture‐recapture dataset (1993–2020) of Indo‐Pacific bottlenose dolphins from the ADS to estimate key population demographic parameters and investigate the effect of climatic factors on dolphin population dynamics. The findings aim to assist in the development of conservation and management strategies.

## 2 Methods

### 2.1 Study Site and Data Collection

**FIGURE 1:**
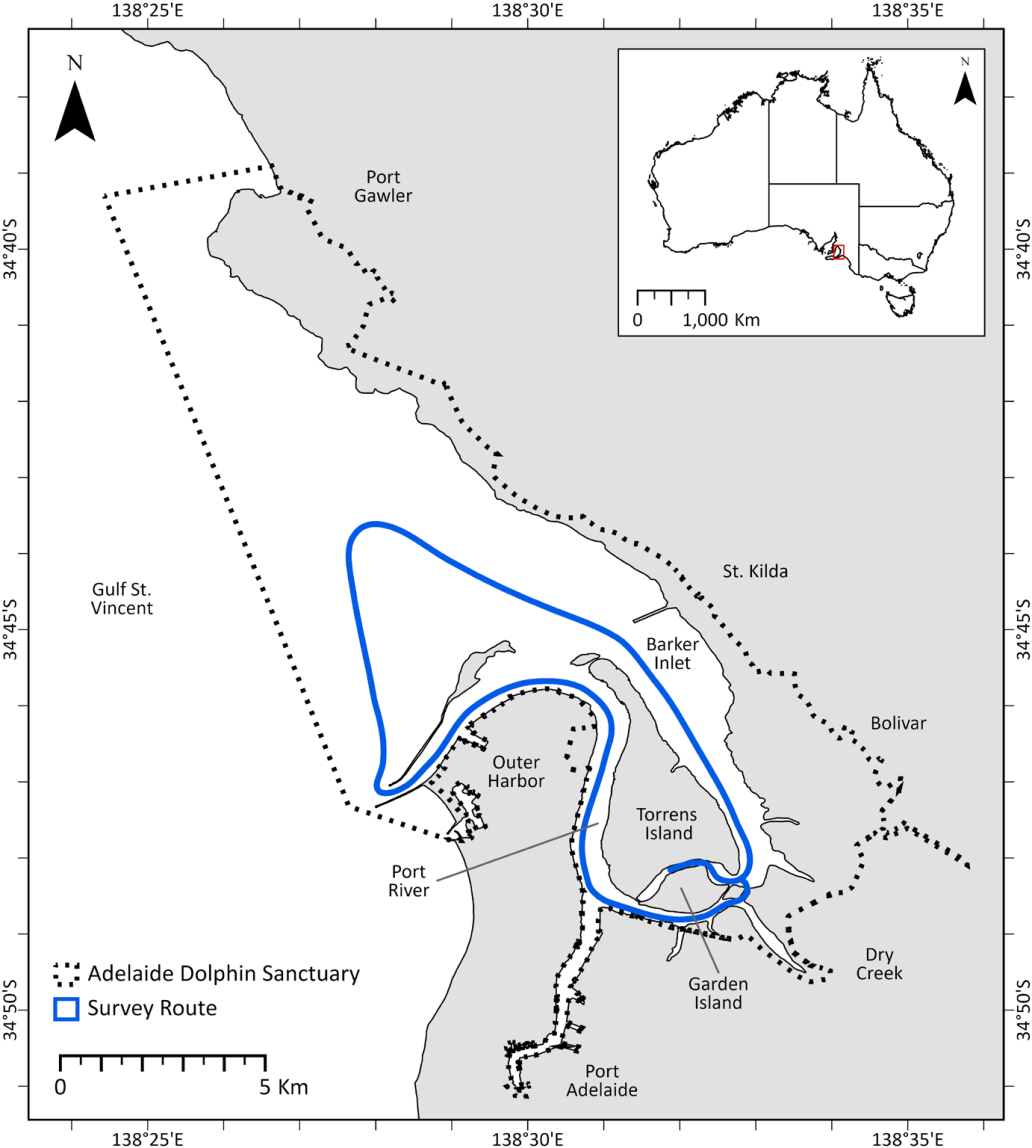
Map of the study area showing the boundary of the Adelaide Dolphin Sanctuary in South Australia and the standard route followed during vessel‐based surveys of Indo‐Pacific bottlenose dolphins ( *Tursiops aduncus* ).

The ADS spans 118 km^2^ along the eastern shore of Gulf St Vincent, South Australia, from Port Gawler Conservation Park to North Haven Marina, and encompasses the Port River and Barker Inlet (Department for Environment and Heritage 2008; Bossley et al. 2017; Figure 1). Boat‐based surveys of bottlenose dolphins following a predetermined route in the ADS were conducted opportunistically year‐round, from January 1989, and are ongoing (Figure 1). The data in this study spans the period January 1993 to December 2020 and includes surveys that covered all or the majority of the survey route. For specific details on the study site and data collection, see Bossley et al. (2017). The age category of each individual dolphin sighted was designated as follows: adults were fully grown animals estimated to be > 1.8 m in length, subadults were dolphins between 1.5 and 1.8 m and not consistently accompanied by an adult, and calves were very small animals ~1 m that were consistently close to an adult (Steiner and Bossley 2008). Calves were excluded until they were identifiable within the population due to their naturally high mortality rate and lack of distinct markings.

### 2.2 Photo ID

Photo‐identification of individual dolphins based on distinctive features of their dorsal fin (e.g., shape, notches, and scars) was used to collect individual capture‐recapture data (Würsig and Würsig 1977). Photographs concentrated on the dorsal fin of individual animals and were taken as perpendicular to the dolphins' body axis as possible. Only photos of sufficient quality to distinguish the distinctive features were used to identify individuals, develop an identification catalogue, and includ in individual capture histories for population analysis (Würsig and Würsig 1977). Dolphins with unique and long‐lasting identifiable features, such as notches and nicks on their dorsal fins, as well as the shape of the dorsal fin, were considered marked. Individuals (typically calves and subadults) lacking persistent distinctive features and observed regularly were followed using marks such as scars or wounds until a persistent mark (notch or nick) developed. Calves of identified mothers regularly observed were followed via their consistent proximity with the mother until they acquired a durable mark. In both cases, the individual was assigned an identification number as soon as the permanent mark occurred, and this number was then retrofitted to the individual in the database prior to the marking event. Sex of adult dolphins was determined by visual observation of the genital area and/or through repeated and consistent observations of an individual in the presence of a dependent calf (for identification of individuals as adult females) (Smolker et al. 1992).

### 2.3 Estimating Abundance, Apparent Survival, and Temporary Emigration

Pollock's Robust Design (PRD) capture‐recapture modelling (Pollock 1982) was used to calculate apparent survival (φ), abundance, and temporary emigration rates (γ”, γ’) using the package RMark (Laake 2013) in the computer program R version 3.6.3 (R Core Team 2013) (see Appendix S1 for further details).

Capture‐recapture histories were compiled for each identified dolphin from 1993– to 2020. Primary periods were defined by Austral Seasons: summer (December–February), autumn (March–May), winter (June–August), and spring (September–November), and secondary periods were defined by the number of months surveyed per season. ‘Transient’ individuals (i.e., individuals that were not encountered again after the first capture between primary periods; Austral seasons) within the population were accounted for using the time‐since‐marking approach (TSM) (Cooch and White 2014; Sandercock 2020). TSM models allow for estimates of apparent survival that are corrected for losses of individuals that are never encountered after the first capture by estimating $\varphi_{1}$ and $\varphi_{2}$ separately (Sandercock 2020) (see Appendix S1 for further information on TSM).

Capture probability (*p*) was set to equal recapture probability (*c*) to help reduce parameter count; given the small study area, size of the dolphin sub‐population, and high resighting rates (as estimated in this study), it is reasonable to assume *p* = *c* (Cooch and White 2014). Capture/recapture probability was set to either constant (.) or dependent on primary or secondary period, group (sex), survey effort, and environmental covariates: rainfall, sea surface temperature (SST), Southern Annular Mode (SAM), and Southern Oscillation Index (SOI). Temporary emigration was modelled as random, Markovian, or absent, while apparent survival estimates were set to constant (.), or to vary by primary period (season), group (sex), and between ‘transients’ and ‘non‐transient’ individuals. *f*0 refers to the number of individuals not seen or ‘missed’ in closed population models. *f*0 relies on capture and recapture probabilities and is a derived parameter estimated from the total population size (N) minus the number of individuals captured at least once during secondary sampling occasions (M) (Figure S1). All possible combinations of models under PRD were run, and the top models were determined using Akaike's Information Criterion (AIC) (Burnham and Anderson 2002).

A locally weighted scatterplot smoothing regression (LOESS) was implemented in R version 4.3.0 (R Core Team 2013) to reveal any underlying trends in abundance estimates across the 27‐year study period (see Appendix S1 for further details). A conservative approach for assigning variance was applied where estimates that had no variance (i.e., abundance estimates equal to the number of individuals captured) were assigned the highest variance obtained from other abundance estimates. A summary fit statistic ($R^{2}$) was derived to assess the goodness of fit of the LOESS curve (Jacoby 2000).

### 2.4 Validation of Model Assumptions and Goodness of Fit

Several assumptions must be met under PRD models to obtain precise estimates of population parameters (Pollock et al. 1990; Pollock 1982; Williams, Nichols, and Conroy 2002); violation of these assumptions can result in biased estimates. Information on the biology of the species as well as goodness‐of‐fit testing were used to validate potential violations of population analyses (Table S1).

### 2.5 Environmental Data for Covariates

To characterise climate conditions and investigate their potential influence on dolphin demographic parameters over time, the following environmental data were obtained to be used as covariates in the PRD analysis: rainfall, SST, SAM, and SOI (see Table S2 for further details).

### 2.6 Proportion of Marked Individuals

Capture‐recapture models can only yield estimates of the number of distinctively marked individuals in a population. This estimate can be adjusted to account for non‐identifiable individuals and provide an estimate of total population size by dividing the estimated abundance of marked dolphins by the proportion of distinctively marked individuals in the population (θ̂) (Wilson, Hammond, and Thompson 1999; Nicholson et al. 2012). $\hat{\theta }$ was estimated using the following formula (Silva et al. 2009; Haughey et al. 2020):

$$\hat{\theta }=\frac{\mathrm{No}.\;\text{of uniquely identified individuals in}\;a\;\text{sighting}}{\text{Total}\;\mathrm{no}.\;\text{of individuals in}\;a\;\text{sighting}}$$

Abundance estimates and confidence intervals of the marked population were adjusted, taking the proportion of marked individuals in the study population into consideration (see Appendix S1 for related formulae).

### 2.7 Site Fidelity

To investigate the tendency of animals to remain in, or return to, and reuse the study area (i.e., site fidelity), we used the total number of recaptures for each individual to calculate the following measures of site fidelity (e.g., Zanardo, Parra, and Möller 2016; Haughey et al. 2020):

1. *Monthly sighting rate*: The number of months a given dolphin was identified as a proportion of the total number of months surveyed.
2. *Seasonal sighting rate/P‐period sighting rate*: The number of seasons (P‐periods) in which a given dolphin was identified as a proportion of the total number of seasons (P‐periods) surveyed.
3. *Yearly sighting rate*: The number of calendar years when a given dolphin was identified as a proportion of the total years surveyed.
4. *Site fidelity indices (SFI)*: The ratio between the number of recaptures for each dolphin and the number of survey months (defined as the number of months between an individual's first capture and its last). A SFI of 1 indicates that an individual was captured on all survey months from its first to its last capture, and a SFI of 0 indicates that it was never recaptured after its first capture (e.g., Simpfendorfer et al. 2011; Zanardo, Parra, and Möller 2016).

Pearson's correlation (*r*) was used to quantify the relationship between the three site fidelity measures (monthly sighting rate, seasonal sighting rate, and yearly sighting rate) using R version 4.3.0 (R Core Team 2013). Site fidelity measures with the lowest correlation were analysed using agglomerative hierarchical clustering (AHC) (Legendre and Legendre 2012) to assess if distinctive groups (or ‘clusters’) of individuals with similar degrees of site fidelity could be identified. AHC analysis was conducted in R version 4.3.0 (R Core Team 2013) using Euclidean distance as the dissimilarity measure. Cophenetic correlation coefficient (CPCC) was used to assess how faithfully clusters in the dendrogram represented the dissimilarities among observations, with a CPCC value > 0.8 indicating a reliable representation of the data (Sokal and Rohlf 1962).

Additionally, the standardised site fidelity index (SSFI) was calculated at the population level following Tschopp et al. (2018). SSFI uses measures of permeance (i.e., the proportion of time spent in the study area from first to last capture over the total number of survey days) and periodicity (i.e., the frequency of recaptures in the study area) to derive a standardised index of site fidelity. The SSFI varies between zero (indicating low site fidelity for the population) and one (indicating high site fidelity for the population) (see Appendix S1 for related formulae).

### 2.8 Residency

Residency patterns (i.e., the amount of time identified individuals stay in the study area) were assessed using lagged identification rates (LIR) (see Appendix S1 for further details). LIRs were calculated only for individuals seen more than once. Varying models of no movement, emigration/mortality, and emigration + mortality were fitted to the observed LIR data (Whitehead 2001). Models were set to 100 bootstraps and AIC corrected for overdispersion (QAICc) was used for model selection (Burnham and Anderson 2002). This analysis was carried out using computer software SOCPROG 2.9 (Whitehead 2001).

## 3 Results

### 3.1 Survey Effort, Photo‐ID, and Proportion of Marked Individuals

Between 1993 and 2020 a total of 1364 surveys were conducted in the ADS, with an average of 48.79 (SD = 19.03, Range = 18–87) surveys conducted per year. On average, 4.06 (SD = 2.54, Range = 1–11) surveys were conducted per month (Table S3). Seasonal survey effort was relatively consistent between 1993 and 2020, with an average of 11.54 (SD = 6.51) surveys conducted in Summer, 13.2 (SD = 6.46) in Autumn, 11.1 (SD = 3.87) in Winter, and 12.9 (SD = 5.23) in Spring.

A total of 14,220 sightings of identified bottlenose dolphins were recorded between 1993 and 2020, out of which 238 dolphins were uniquely identified (35 males, 65 females, and 138 of unknown sex). Most identified individuals (53%) were first seen as adults (assumed to be born at least 10 years before or more), and the rest as they transitioned from subadult to adult (assumed to be born 10 years before) or observed as a calf (birth date known, and therefore age known). Between 1993 and 2020, 25 individuals are known to have died (body collected or observed deceased), 11 of whom were female and 14 were male.

**FIGURE 2:**
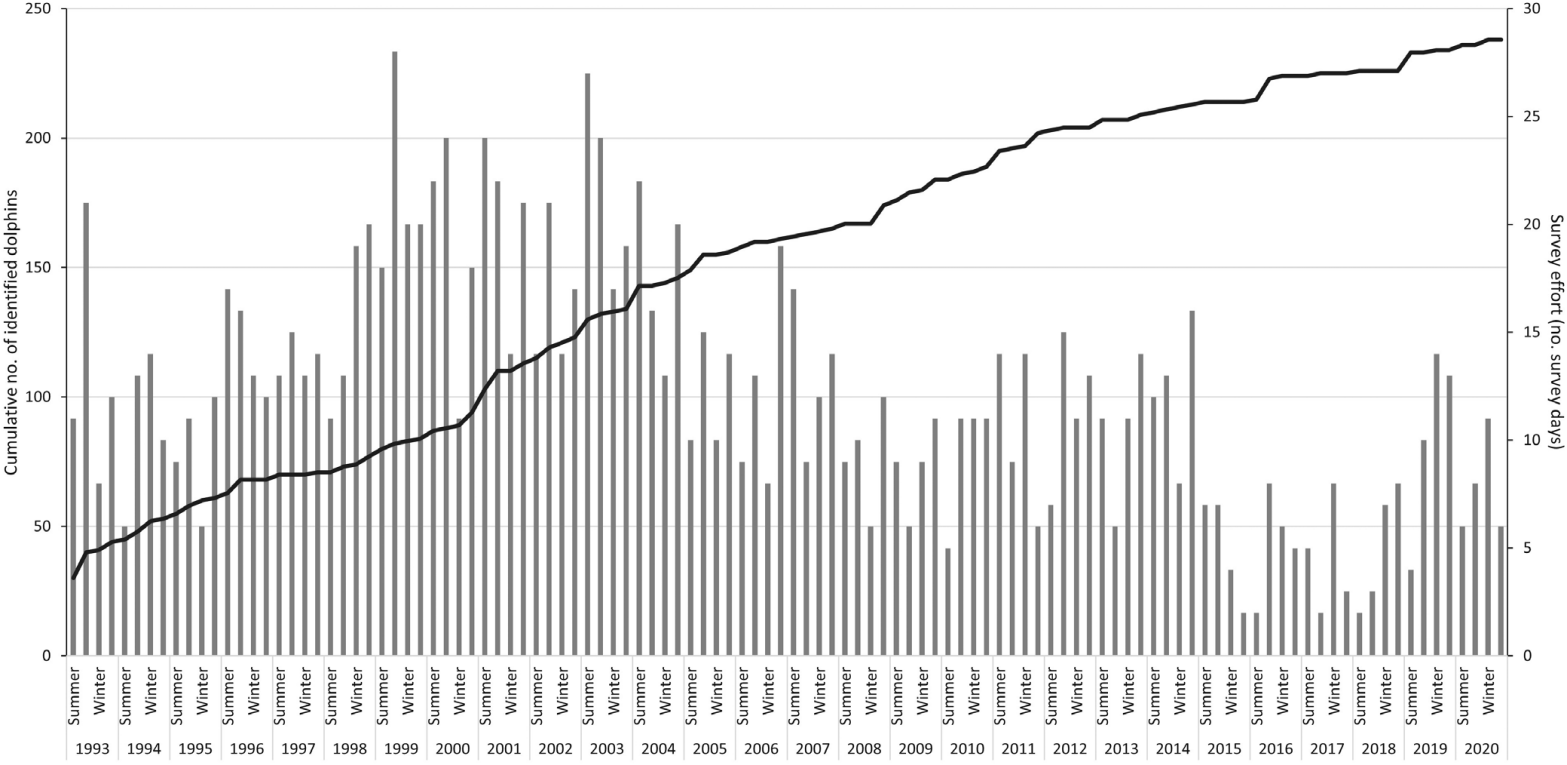
Cumulative discovery curve of identified Indo‐Pacific bottlenose dolphins ( *Tursiops aduncus* ) in the Adelaide Dolphin Sanctuary, South Australia, over the Austral seasons between 1993 and 2020. Columns represent the number of survey days per season.

The cumulative discovery curve of newly marked individuals per season increased gradually over the 27‐year study period, indicating that, overall, new individuals were continually sighted within the study area (Figure 2). When seasons are broken down into months, the discovery curve plateaus across some years, suggesting that during this time the study area was mostly used by previously identified individuals (Figure S2).

The proportion of marked individuals within the study population ($\hat{\theta }$ ± SE) was estimated at 0.60 ± 0.03.

### 3.2 Goodness of Fit

Goodness‐of‐fit (GOF) tests from U‐CARE provided a variance inflation factor of $\hat{c}$ = 2.46 (Table S4). $\hat{c}$ values of 2–3 are typical for large capture‐recapture datasets with several individuals (Choquet et al. 2009). While there is no definitive rule stating that overdispersion causes a lack of fit of the data to the model, a general rule is that $\hat{c}$ should not exceed 3–5 (Choquet et al. 2009; Burnham and Anderson 2002). Small structural effects that result in an overall $\hat{c}$ of 1–3 can be treated as ‘noise’ (Choquet et al. 2009). See Appendix S1 for further details.

### 3.3 Pollock's Robust Design

A total of 198 model variations were run under Pollock's Robust Design (PRD). The top model supported by AIC had apparent survival (φ) varying between ‘transients,’ females, males, and individuals of unknown sex, while remaining constant for these groups between primary periods (seasons), Markovian temporary emigration varying by season, capture probability varying with survey effort, and the number of individuals not seen (*f*0) varying by season (Table 1). The top model accounted for 90% of the AIC weight. Environmental covariates (rainfall, SST, SAM, and SOI) did not have a direct influence on the demographic parameters estimated.

**TABLE 1:** Summary of the top 10 most supported Pollock's robust design models fitted to mark‐recapture histories of Indo‐Pacific bottlenose dolphins ( *Tursiops aduncus* ) in the Adelaide Dolphin Sanctuary, South Australia, from 1993 to 2020. Models were used to estimate abundance (Nm), apparent survival (φ), temporary emigration (γ”, γ’), capture and recapture probability (*p* = *c*), and number of individuals not seen (*f*0). The notion (.) indicates that a given parameter was kept constant. ‘Group’ refers to individual sex (female, male, or unknown), ‘transient’ refers to individuals that were never encountered after first capture between primary periods (Austral seasons), and effort refers to survey effort [i.e., number of survey days per secondary period (months)]. The top model is shown in bold.

Model	No. par.	AICc	▲AICc	Weight	Deviance
φ **(transients + group), γ”(season) ≠ γ’(season), *p* = *c*(effort), *f*0 (season)**	**336**	**10573.5**	**0.00E+00**	**9.00E‐01**	**25536.82**
φ(season), γ”(season) ≠ γ’(season), *p* = *c*(effort), *f*0(season)	332	10577.89	4.40E+00	1.00E‐01	25549.93
φ(.), γ”(season) ≠ γ’(season), *p* = *c*(effort), *f*0(season)	274	10599.63	2.61E+01	1.90E‐06	25696.99
φ(transients + group), γ”(season) ≠ γ’(season), *p* = *c*(effort), *f*0(month)	226	10637.78	6.43E+01	9.91E‐15	25837.44
φ(group), γ”(.) ≠ γ’(season), *p* = *c*(effort), *f*0(season)	225	10696.24	1.23E+02	0.00E+00	25898.02
φ(transients + group), γ”(.) ≠ γ’(season), *p* = *c*(effort), *f*0(month)	108	10698.15	1.25E+02	0.00E+00	26144.02
φ(transients + group), γ”(season) ≠ γ’(.), *p* = *c*(effort), *f*0(season)	210	10711.35	1.38E+02	0.00E+00	25944.83
φ(group), γ”(season) ≠ γ’(season), *p* = *c*(effort), *f*0(season)	334	10729.85	1.56E+02	0.00E+00	25697.53
φ(.), γ”(season) ≠ γ’(season), *p* = *c*(effort), *f*0(month)	223	10746.9	1.73E+02	0.00E+00	25952.91
φ(group), γ”(.) ≠ γ’(season), *p* = *c*(effort), *f*0(month)	108	10753.24	1.80E+02	0.00E+00	26199.1

### 3.4 Abundance Estimates

**FIGURE 3:**
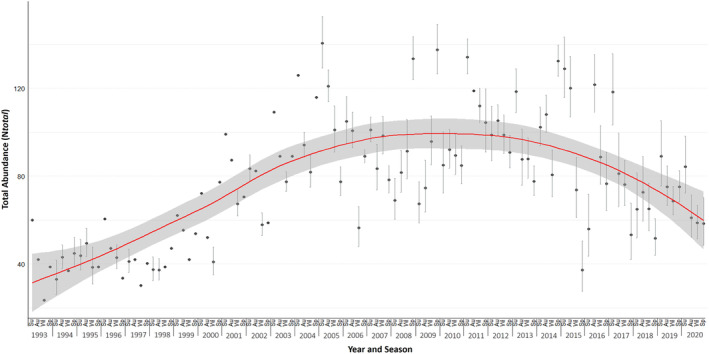
Locally weighted scatterplot smoothing regression (LOESS) was applied to abundance estimates of Indo‐Pacific bottlenose dolphins ( *Tursiops aduncus* ) in the Adelaide Dolphin Sanctuary, South Australia, between 1993 and 2020 (Summer = Su, Autumn = Au, Winter = Wi, and Spring = Sp). Dark‐grey circles represent abundance estimates, light‐grey lines represent upper and lower confidence intervals, and the solid red line represents the underlying trend of the datapoints weighted by the variance.

Estimates of abundance (*N*_total_) varied seasonally and, as indicated by the top PRD model, were linked to seasonal movements of individuals into and out of the study area. Abundance estimates across the study period (1993–2020) were highest in summer with an average estimate of 85.37 individuals present in the study area and lowest in winter with an average abundance estimate of 68.57 individuals. The lowest abundance of dolphins in the study area was 23.51 in winter 1993, and the highest was 140.44 in summer 2005 (Figure 3).

On average there were 30.91 females using the ADS, 23.63 males, and 21.68 dolphins of unknown sex between 1993 and 2020. Among the identifiable individual dolphins of known sex, females had consistently higher abundance estimates than males across the study period, particularly in summer (*N*_total_ = 33.90; SE 2.38) and autumn (*N*_total_ = 32.11; SE 2.14; Table 2). Male abundance remained, on average, comparable throughout all seasons, while female abundance was lowest in winter and highest in summer (Table 2).

**TABLE 2:** Average seasonal estimates of abundance of female, male, and unknown sex Indo‐Pacific bottlenose dolphins ( *Tursiops aduncus* ) in the Adelaide Dolphin Sanctuary, South Australia, from 1993 to 2020. Abundance estimates (*N*_total_) have been adjusted to account for unmarked individuals by calculating the proportion of identified individuals ($\hat{\theta }$).

Season	Female			Male			Unknown		
	*N*_total_	SE	95% CI	*N*_total_	SE	95% CI	*N*_total_	SE	95% CI
Summer	33.90	2.38	29.02–38.78	24.38	1.04	22.25–26.50	26.66	2.91	20.68–32.63
Autumn	32.11	2.14	27.72–36.50	24.61	0.81	22.95–26.27	22.34	2.60	17.00–27.68
Winter	26.59	1.95	22.59–30.59	23.14	0.99	21.12–25.17	18.46	2.25	13.84–23.08
Spring	31.04	2.51	25.90–36.18	22.41	0.985	20.24–24.35	19.94	2.72	14.35–25.52

A locally weighted scatterplot smoothing regression analysis indicated an increasing trend in abundance during the early years of the study, from 1993 (*N*_total_ = 20–40 individuals) to (approximately) 2002 (*N*_total_ = upwards of 40–50 individuals) (Figure 3). This is followed by a plateau between 2003 and 2016 where abundance estimates were relatively high (*N*_total_ = 100+ individuals) (see Table S5 for all abundance estimates). From 2011 onwards, the LOESS trendline shows a steady decline, with abundance estimates decreasing to levels similar to the early 2000s (Figure 3). It is important to note that the conservative approach of assigning the highest variance to datapoints with missing variance values may have contributed to the variability in the goodness‐of‐fit of the LOESS regression, resulting in a moderate summary fit statistic ($R^{2}$ = 0.57).

### 3.5 Apparent Survival and Temporary Emigration

The top model selected by AIC suggests that apparent survival (φ) varies between ‘transients’ (i.e., individuals seen only once per season) and females, males, and individuals of unknown sex but remains constant from season to season for each group. Apparent survival estimates for females, males, and dolphins of unknown sex seen more than once were higher than those of ‘transient’ individuals. Male and female apparent survival were high and consistent across seasons, whereas dolphins of unknown sex showed slightly lower apparent survival (Table 3).

**TABLE 3:** Estimates of apparent survival (φ) from the best fitting Pollock's Robust Design mark‐recapture model for Indo‐Pacific bottlenose dolphins ( *Tursiops aduncus* ) in the Adelaide Dolphin Sanctuary, South Australia, between 1993 and 2020. Estimates for the ‘non‐transient’ individuals seen more than once per season are shown in bold.

Sex	φ	SE	95% CI
Female	0.889	0.020	0.852–0.929
	**0.990**	**0.001**	**0.988–0.993**
Male	0.881	0.022	0.839–0.925
	**0.990**	**0.002**	**0.986–0.993**
Unknown	0.760	0.030	0.702–0.821
	**0.976**	**0.003**	**0.970–0.981**

The PRD model most supported by AIC suggests that temporary emigration is Markovian and varies by season. The probability of an individual emigrating out of the study area if it was previously seen (γ”) was lower than the probability of an individual staying out of the study area (γ’) (Table 4). Temporary emigration (γ”) was highest between autumn and winter, and winter and spring. The probability of individuals remaining out of the study area (γ’) was higher between winter and spring (Table 4). For a full table of temporary emigration estimates, see Table S6.

**TABLE 4:** Average estimates of Markovian temporary emigration (γ”, γ’) from the best‐fitting Pollock's Robust Design mark‐recapture model for Indo‐Pacific bottlenose dolphins ( *Tursiops aduncus* ) in the Adelaide Dolphin Sanctuary, South Australia, between 1993 and 2020. γ” is the probability of being a temporary emigrant if an animal was present in the previous primary period (season), and γ’ is the probability of being a temporary emigrant if an animal was absent in the previous primary period (i.e., remaining out of the study area).

Season	Average γ”	SE	95% CI	Average γ’	SE	95% CI
Autumn–Winter	0.19	0.02	0.15–0.23	0.77	0.04	0.68–0.86
Winter–Spring	0.20	0.02	0.16–0.24	0.89	0.02	0.85–0.93
Spring–Summer	0.11	0.02	0.07–0.15	0.75	0.04	0.68–0.83
Summer–Autumn	0.13	0.02	0.08–0.17	0.66	0.04	0.57–0.75

### 3.6 Site Fidelity

**FIGURE 4:**
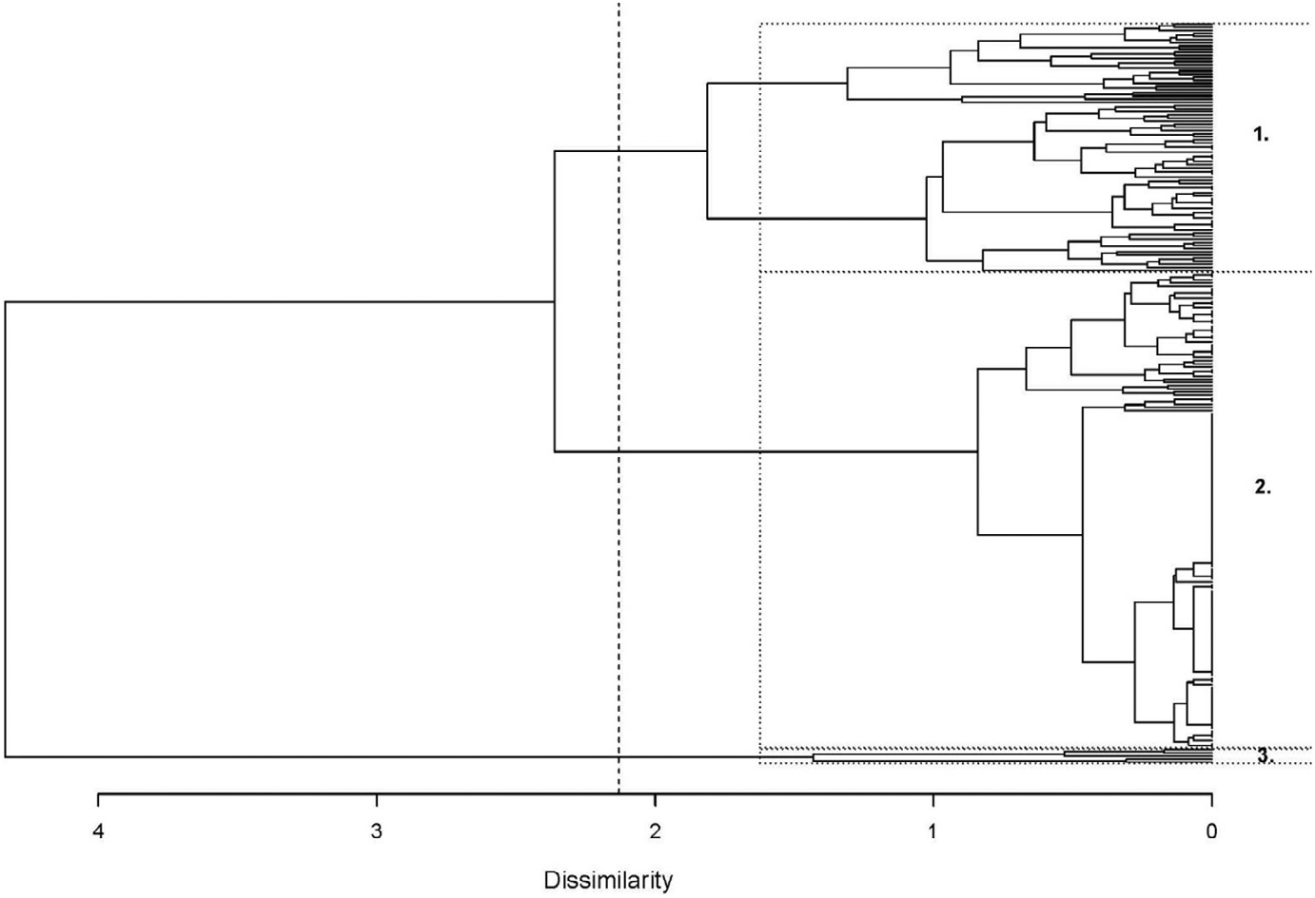
Dendrogram of agglomerative hierarchical clustering (AHC) analysis of Indo‐Pacific bottlenose dolphins ( *Tursiops aduncus* ) in the Adelaide Dolphin Sanctuary based on site fidelity patterns. Rectangles represented by dotted lines indicate three clusters/groups: Group 1 (‘residents’, *n* = 80), Group 2 (‘occasional visitors’, *n* = 153), and Group 3 (‘long‐term residents’, *n* = 5). The dashed line represents the dissimilarity threshold = 2.13.

Monthly and yearly sighting rates between 1993 and 2020 indicated that uniquely identified individuals were resighted in the study area for a period of approximately 3–7 years on average (Table 5). Identified females and males shared similar yearly sighting rates, which were higher compared to individuals of unknown sex. Males also had a higher seasonal sighting rate and were seen in 41 out of 112 seasons surveyed between 1993 and 2020 compared to females, who were only seen in 29 seasons (Table 5). The standardised site fidelity index (SSFI) for the total population was estimated at 0.03, with males showing the highest SSFI at 0.09. Seasonal sighting rates correlated strongly with monthly (*r* = 0.97) and yearly (*r* = 0.93) sighting rates and thus were removed from AHC analysis. AHC analysis based on monthly and yearly sighting rates (*r* = 0.84) identified three main clusters (dissimilarity threshold = 2.13) (Figure 4). The high cophenetic correlation coefficient (CPCC = 0.85) confirmed the dendrogram's reliability in representing dissimilarities among observations. Cluster 1 (*n* = 80) consisted of ‘resident’ individuals seen in over half of all survey years (mean yearly sighting rate = 0.57, SD = 0.15) and in over 70 of all 318 survey months. Cluster 2 (*n* = 153) was the largest cluster and consisted of ‘occasional visitors’ seen on average 3 years out of 27 survey years (yearly sighting rate 0.12, SD = 0.09) and in approximately 6 months out of the 318 survey months. Cluster 3 was the smallest (*n* = 5) and included ‘long‐term residents’ seen in almost all 27 survey years (yearly sighting rate 0.80, SD = 0.14) and in 206 months out of all 318 survey months.

**TABLE 5:** Average sighting rates, site fidelity indices (SFI), and recaptures for Indo‐Pacific bottlenose dolphins ( *Tursiops aduncus* ) in the Adelaide Dolphin Sanctuary, South Australia, between 1993 and 2020.

	Monthly (±SD)	Yearly (±SD)	Seasonal (±SD)	SFI (±SD)	No. recaptures (±SD)
Female	0.15 (±0.15)	0.43 (±0.25)	0.26 (±0.21)	0.47 (±0.51)	85.68 (±114.98)
Male	0.26 (±0.19)	0.45 (±0.25)	0.37 (±0.23)	1.60 (±2.54)	167.86 (±145.62)
Unknown Sex	0.04 (±0.09)	0.17 (±0.19)	0.08 (±0.14)	0.18 (±0.29)	20.11 (±44.81)
Total	0.11 (±0.15)	0.28 (±0.25)	0.17 (±0.21)	0.47 (±1.14)	59.74 (±102.72)

### 3.7 Residency

**FIGURE 5:**
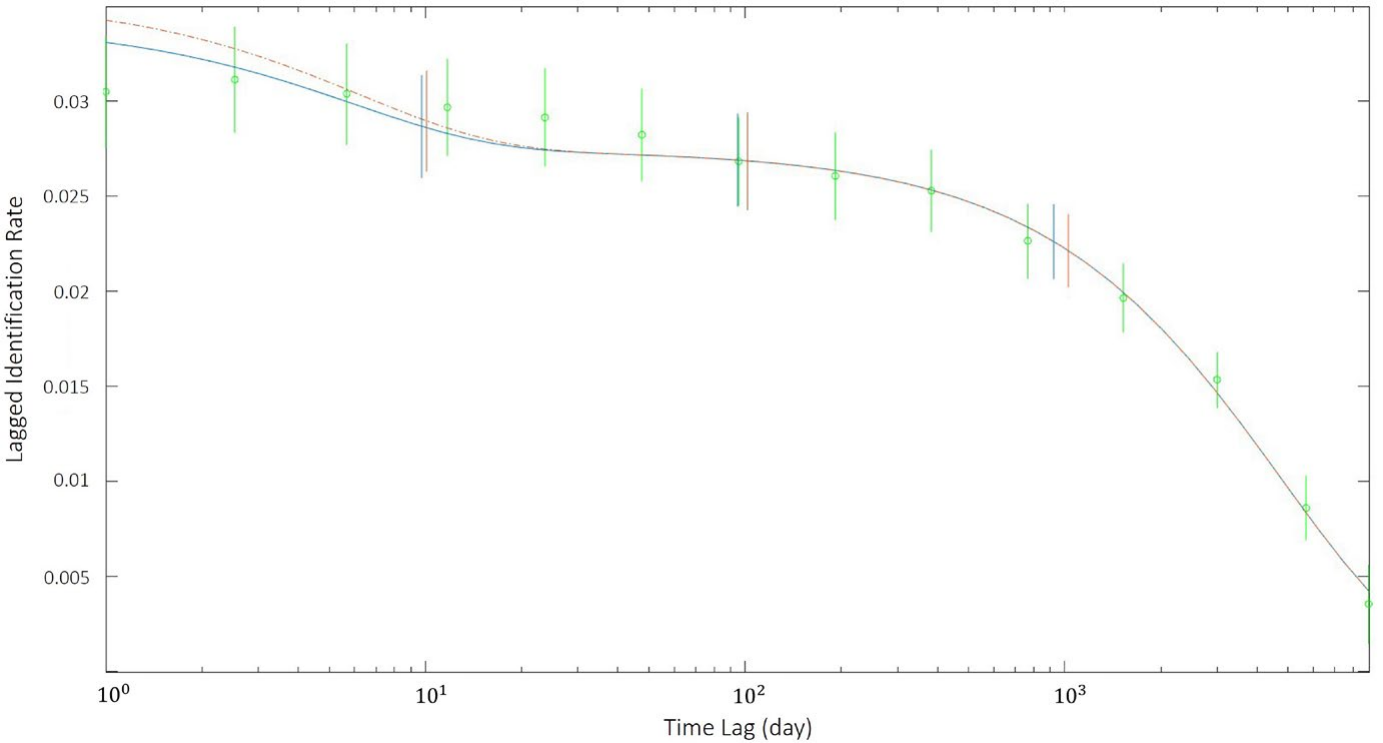
Lagged identification rates (LIR) of Indo‐Pacific bottlenose dolphins ( *Tursiops aduncus* ) in the Adelaide Dolphin Sanctuary, South Australia. Data points are represented by circles and fitted with two parametrised versions of the best‐fit model (emigration + reimmigration + mortality).

LIR dropped between 0 and 10 days, indicating that some individuals spend a short amount of time within the study area (Figure 5). LIR remained relatively stable between 10 and 1000 days (equivalent to approximately 3 years) and did not level off above zero, suggesting that some animals are ‘residents’ to the area during this time. After 1000 days, LIR drops, most likely due to permanent emigration and/or mortality as identified by the top models (Table 6). Of the models applied to the data, the model of emigration + reimmigration + mortality fit the data best. This model estimated that there are about 28.22 dolphins in the study area at any one time, a mean residency time inside the study area of 27.45 days, and a mean time spent outside the study area of 8 days.

**TABLE 6:** Models fitted to observed lagged identification rate (LIR) data for Indo‐Pacific bottlenose dolphins ( *Tursiops aduncus* ) in the Adelaide Dolphin Sanctuary, South Australia, based on monthly and yearly sighting rates. For a description of model equations, see (Whitehead 2001). The model(s) best fitted to the data according to Akaike's Information Criterion (AIC), corrected for overdispersion (QAICc), are shown in bold. ΔQAICc indicates how well the data support the less favoured model (Burnham and Anderson 2002).

Model equation	Model explanation	QAICc	ΔQAICc
**a3 × exp(−a1 × td) + a4 × exp(−a2 × td)**	**Emigration + reimmigration + mortality**	**13479012.5203**	
**(exp(−a4 × td)/a1). × ((1/a3) + (1/a2) × exp(−(1/a3 + 1/a2) × td))/(1/a3 + 1/a2)**	**Emigration + reimmigration + mortality; a1 = N; a2 = Res time in; a3 = Res time out; a4 = Mort**	**13479013.2777**	**0.76**
(1/a1) **×** exp.(−td/a2)	Emigration/mortality; a1 = N; a2 = Mean residence	13479075.8802	63.36
a2 **×** exp.(−a1 **×** td)	Emigration/mortality	13479075.8803	63.36
(1/a1) **×** ((1/a3) + (1/a2) **×** exp.(−(1/a3 + 1/a2) **×** td))/(1/a3 + 1/a2)	Emigration + reimmigration; a1 = N; a2 = Res time in; a3 = Res time out	13479077.8802	65.36
a2 + a3 **×** exp.(−a1 **×** td)	Closed: emigration + reimmigration	13685791.9348	206779.41
a1	Closed	13686101.6238	207089.10
1/a1	Closed	13686101.6238	207089.10

## 4 Discussion

Understanding variation in population demographic parameters of threatened species living amidst urbanisation and climate change is crucial for their effective conservation and management. This study highlights the significance of the ADS as a habitat for Indo‐Pacific bottlenose dolphins within the Gulf St Vincent despite being a highly urbanised environment. Demographic estimates reveal seasonal changes in dolphin abundance within the ADS, which peaks in summer (range = 33–140 individuals) and is lowest in winter (range = 24–112 individuals), with many dolphins regularly returning to the area for multiple years. Total population estimates accounted for the proportion of marked individuals in the study area ($\hat{\theta }$ = 0.60 ± 0.03) to minimise bias introduced by unmarked individuals. A low proportion of marked individuals can introduce uncertainty into demographic estimates; however, in small populations like the ADS, a marked population of 60% is relatively high and provides a strong basis for demographic estimates to be representative of the population in the study area, provided assumptions of homogeneity remain (i.e., marked individuals move, behave, and have similar survival risks to unmarked individuals).

Estimates of dolphin abundance in the ADS were not directly related to environmental variables (rainfall, SST, SAM, and SOI) but were linked to seasonal temporary emigration movements. Global and local weather patterns can play a crucial role in shaping the demographic patterns of wildlife populations by influencing survival, reproduction, habitat conditions, and interspecies interactions (Stenseth et al. 2002). Identifying relevant drivers of demographic change is challenging because there may be multiple candidate variables, time lags between environmental events and demographic responses, differential influences on sexes and age classes, and indirect effects mediated through interactions with other species (Stenseth et al. 2002). Whilst we used local and global climate variables that have been shown to influence bottlenose dolphin population demographic parameters elsewhere (Sprogis et al. 2018), the results here indicate that there may be other more influential climatic and/or biological factors involved in the ADS dolphin's population demographics.

Calving season in the ADS occurs between summer and autumn, with 89% of calves typically born throughout December–April, coinciding with warmer SST (Steiner and Bossley 2008; Crook et al. 2020). Calving has previously been linked to warmer water temperatures in bottlenose dolphins, as warm water is thermally efficient for small calves that have a thinner blubber layer and, thus, a lesser ability to maintain their body heat (Mann et al. 2000; Daura‐Jorge et al. 2005). The study area also offers a higher level of protection from predators, as it is a relatively enclosed habitat with shallow waters that support dolphin survival, particularly during times of reproduction when there are young calves present. Due to the 12‐month gestation of this species, peak mating and calving periods usually overlap. The higher estimates of dolphin abundance and lower temporary emigration rates observed in summer are, therefore, likely a result of individuals preferring the warm, shallow, and protected waters of the study area during peak mating/calving season. These findings are supported by Bossley et al. (2022), who found a clear seasonal pattern of dolphin sightings in the ADS, with more sightings during the warmer months. Such seasonal variations in demographic estimates have also been found in other coastal dolphin populations around Australia and the world (e.g., Hubard et al. 2004; Smith et al. 2016; Zanardo, Parra, and Möller 2016).

Another important biological factor that may be driving seasonal patterns in dolphin demographics in the ADS is changes in prey availability. Marine mammal prey are ectothermic; thus, their distribution is largely driven by their external environment (Bearzi, Notarbartolo‐Di‐Sciara, and Politi 1997; Lin, Akamatsu, and Chou 2015). Higher estimates of dolphin abundance and reduced movement out of the study area throughout summer may be linked to increased prey availability within and around the estuary during the warmer months. The Port River estuary is known to be an important nursery area for both commercially and recreationally caught fish (Connolly 1994; Jackson and Jones 1999; Jones et al. 1996), and whilst there are limited recent data on prey abundance in the ADS, an influx of prey species is known to occur during summer in the Adelaide metropolitan waters adjacent to the ADS (Bryars 2003; Rogers, Dimmlich, and Ward 2008). The seasonal abundance of bottlenose dolphins in these waters has previously been linked to the seasonally driven fluctuations in prey availability (Zanardo, Parra, and Möller 2016), and it is likely that a similar pattern of seasonally fluctuating abundance estimates of dolphins in the ADS results from a degree of connectivity between the Port River estuary and the adjacent metropolitan waters in Gulf St Vincent.

Estimates of temporary emigration also suggest that most individuals range beyond the limits of the study area, which only encompasses the inner waters of the ADS. Dolphins are often sighted in the outer area of the ADS and in adjacent waters of Gulf St Vincent; thus, it is likely that dolphins identified in the current study are moving between these areas and the current study site. This is supported by our site fidelity results, which suggest that site fidelity at the population level is low due to the large number of individuals (*n* = 153) that ‘occasionally visit’ the study area as identified through AHC analysis. Consequently, population estimates in this study may not be representative of the entire ADS population, as they are only based on individuals sighted within the Port River—Barker Inlet portion of the ADS. Future studies incorporating complementary techniques (e.g., distance sampling) and covering the entire ADS could further validate and enhance population estimates. Despite having low site fidelity at the population level, estimates of LIR revealed that the ADS typically hosts a population of approximately 28 individuals at any one time. The high volume of ‘occasional visitors’ to the ADS, in addition to those that have regularly returned to and utilised the study site across the 27‐year study period, with some remaining resident to the area, highlights the significance of this habitat to the broader population of Indo‐Pacific bottlenose dolphins in Gulf St Vincent.

Despite the large number of dolphins that regularly visit and reuse the study site and the increasing trend in abundance during the early 2000s, our trend analysis revealed a recent decline in dolphin abundance, which may be signalling towards a maladaptive population response. Improvements in water quality may explain the increasing trend in abundance estimates during the early 2000s (Bossley et al. 2017), and since then, the higher estimates of dolphin abundance have likely been maintained thanks to conservation efforts following the implementation of the ADS in 2005. However, in ecosystems with consistently high levels of human impact, such as the ADS, individuals can become habituated to disturbance and continue to use an affected but still beneficial habitat—this is known as ‘synurbanisation’ (Santini et al. 2019). Bottlenose dolphins have great behavioural plasticity, which allows them to adapt to altered environmental conditions and exhibit remarkable resilience, with many populations managing to persist and, in some cases, thrive in areas heavily impacted by humans (Bearzi, Piwetz, and Reeves 2019). This resilience has previously been demonstrated in the ADS, where dredging activities in the Port River estuary did not affect the long‐term occurrence of the dolphins (Bossley et al. 2022). Other examples include in Charleston Harbour, South Carolina, where the consistent presence of large ships allows dolphins to herd fish along the sides of docked ships (Weinpress‐Galipeau et al. 2021). Similarly, bridge construction in Florida saw common bottlenose dolphins adapting the timing of their behaviours within the construction zone to later in the day when construction activities were minimised (Weaver 2021). Whilst this behavioural plasticity may compensate for some of the potential harm, it can also be maladaptive and involve risk to health or drive long‐term evolutionary change (Bearzi, Piwetz, and Reeves 2019; Ritzel and Gallo 2020). Further research is needed to understand the cumulative impacts triggering the dolphin population decline in this heavily urbanised area and to assess the future viability of this population.

### 4.1 Implication for Conservation

Adult survival and reproduction have been found to be most important for the viability of slow‐growing populations, characterised by late maturation, small litter size, and long life spans, such as bottlenose dolphins (Manlik et al. 2016). Given the high and stable apparent survival (0.98–0.99) found in this study for ADS dolphins, and the relatively high first‐year calf mortality (30%) and pre‐weaning mortality (46%) reported for this population (Steiner and Bossley 2008), management and conservation strategies aimed at increasing reproductive rates are likely to be more effective at reversing population declines. Dolphins can alter their behaviour and movement in response to vessel traffic and vessels moving at high speeds, including a reduction in the frequency of socialising/mating behaviours (reviewed in Mills, Piwetz, and Orbach 2023). Thus, restrictions on vessel traffic and speed limits in identified dolphin's core areas of use in the ADS, such as Angas Inlet (Garden Island), Light Passage (Outer Harbour), and Port River/North Arm Junction (Newman et al., in prep.) during warmer months (Dec–Feb), when dolphin abundance is highest and calving season is at its peak, could have a positive effect on mating behaviour, reproduction, and calf survival. Future research should be aimed at assessing the importance of survival and reproduction for ADS dolphin population viability and the effectiveness of different management scenarios in increasing reproductive rates and/or increasing survival.

Connectivity is fundamental to the health and persistence of animal populations and the ecosystems they inhabit (Cowen et al. 2007). The seasonal fluctuations of dolphin abundance and temporary emigration estimates in the ADS suggest connectivity to adjacent waters that future conservation and management practices should work to maintain. Increased coastal development in the area and noise pollution from increased shipping and construction can interfere with dolphin communication, disrupting their natural social interactions and movement. Increased human activity and poor ecosystem health in the ADS may deter and/or prevent dolphins in extended areas of Gulf St Vincent from visiting and using the area. Conservation strategies that work towards reducing anthropogenic disturbance in the area, as well as improving ecosystem health, should be prioritised to promote connectivity to adjacent waters and dolphin populations.

## 5 Conclusion

Indo‐Pacific bottlenose dolphins have been using, visiting, and residing in the ADS for over three decades. Results from this long‐term study reveal the seasonal variation of abundance and movement into and out of the study area is not directly related to environmental variables representative of large‐scale climatic events. Demographic fluctuations for dolphins in this area are likely related to biological factors, such as reproduction and prey availability. Estimations of temporary emigration, in addition to low site fidelity at the population level, suggest that most individuals are using areas outside of the study area. Future studies should assess broader areas of the Sanctuary and Gulf St Vincent to better understand population demographics and connectivity to adjacent dolphin groups, communities, or populations. Future research should also work towards a multidisciplinary approach to provide fine‐scale, site‐specific data to help further assess drivers of ADS dolphin population demographics. The ADS and Port River estuary has a longstanding history of human disturbance with some impacts that have likely accumulated over time, the effects of which may only be apparent in recent years. It is important that future management and conservation strategies consider the impacts of historical cumulative threats and ensure that further pressures are not added to an already impacted ecosystem.

## Author Contributions

**Kennadie Haigh:** conceptualization (equal), formal analysis (lead), writing – original draft (lead), writing – review and editing (equal). **Guido J. Parra:** conceptualization (equal), supervision (lead), writing – review and editing (equal). **Luciana Möller:** conceptualization (equal), supervision (supporting), writing – review and editing (equal). **Aude Steiner:** data curation (supporting), writing – review and editing (equal). **Mike Bossley:** data curation (lead), writing – review and editing (equal).

## Conflicts of Interest

The authors declare no conflicts of interest.

## Supporting information


Appendix S1:

## Data Availability

The data underlying this article cannot be shared publicly due to legal restrictions. We have a legal obligation under the terms of the agreement with the author of the raw data (MB). This obligation prohibits us from publishing or making the raw data publicly available.
