# Supplementary material for: Long‐Term Demographic Trends of Near Threatened Coastal Dolphins Living in an Urban Estuary
Source: Ecol Evol. 2025 Jan 6;15(1):e70834. doi: 10.1002/ece3.70834 (PMC11705426; doi:10.1002/ece3.70834)
Supplement: Supplementary file 1 — Appendix S1: [file ECE3-15-e70834-s001.docx]

## Methods

### 1.1 Estimating abundance, apparent survival, and temporary emigration

#### Pollock’s Robust Design

Pollock’s Robust Design (PRD) combines closed and open population models under a nested sampling framework. This includes primary periods that are long enough to allow for population gains through births and immigrations, and losses from death and emigration, and secondary periods that are sufficiently shorter so that no gains or losses are assumed to occur (Pollock et al., 1990). Closure is assumed within secondary and primary periods but not between primary periods. PRD allows probability of first capture (p), recapture (c) to be estimated, and results in derived parameter estimates of abundance (N) and the number of individuals not captured or ‘missed’ (*f*0) to be estimated for each secondary sampling period. The estimation of *f*0 combined with observed captures (M) assists broader analyses at primary occasions where apparent survival ($\varphi$) and temporary emigration (γ”, γ’) are estimated between primary sampling periods (Kendall et al., 1997, Pollock, 2000, Cooch and White, 2014). To clarify:

If

$$N=M+ f0$$

Then *f*0 is estimated as

$$f0=N-M$$

PRD was chosen for analysis as it represents a biologically realistic approach to the analysis of capture-recapture data over long periods of time for the population in question, and also allows the use of individual and group covariates to help identify variation in parameter estimates (Kendall et al., 1997, Cooch and White, 2014).

Estimating demographic parameters of highly mobile species is difficult, particularly when populations contain ‘non-resident’ or ‘transient’ individuals (Haughey et al., 2020). In populations where transient individuals are present, apparent survival of newly marked individuals will be lower in the interval after first capture $\varphi_{1}$than returning individuals detected in the following interval $\varphi_{2}$ (Sandercock, 2020). To overcome this challenge, transients within the population were accounted for using the time-since-marking approach (Cooch and White, 2014, Sandercock, 2020). TSM models allow for estimates of apparent survival that are corrected for losses of individuals that are not encountered after the first capture by estimating $\varphi_{1}$ and $\varphi_{2}$ separately (Sandercock, 2020).

$$\left\{ \begin{aligned} \varphi(.) \\ \varphi(season) \\ \varphi(group) \\ \varphi(transients+group) \\ \\ \end{aligned} \right\}\times\left\{ \begin{aligned} \boldsymbol{NO EMIGRATION} \\ \gamma"=\gamma'=0 \\ \boldsymbol{RANDOM EMIGRATION} \\ \gamma"(.)=y'(.) \\ \gamma"(season)=y'(season) \\ \boldsymbol{MARKOVIAN EMIGRATION} \\ \left( \begin{aligned} \gamma"(.) \\ \gamma"(season \end{aligned} \right)\boldsymbol{\times}\left( \begin{aligned} \gamma^{'}(.) \\ \gamma^{'}\left( season \right) \end{aligned} \right) \\ \end{aligned} \right\}\times\left\{ \begin{aligned} p=c\left( . \right) \\ p=c\left( month \right) \\ p=c\left( season \right) \\ p=c\left( group \right) \\ p=c(effort) \\ p=c\left( rainfall \right) \\ p=c\left( SST \right) \\ p=c\left( SOI \right) \\ p=c\left( SAM \right) \end{aligned} \right\}\times\left\{ \begin{aligned} f0 (season) \\ f0(month) \end{aligned} \right\}$$

**Apparent Survival**

**Movement**

**Capture and recapture probabilities**

**Figure S1:** Pollock's Robust Design (PRD) model parameters considered for assessment of population demographics of Indo-Pacific bottlenose dolphins *(Tursiops aduncus)* in the Adelaide Dolphin Sanctuary, South Australia. The models are represented as different combinations of the parameter specification (198 model variations total). The notation (.) indicates a given parameter was kept constant, ‘season’ indicates that a parameter was allowed to vary with Austral season, ‘group’ indicates that a parameter was allowed to vary by sex (female, male, or unknown sex), ‘transient + group’ indicates that a parameter was allowed to vary between individuals that were not encountered after their first capture (transients) and individuals seen more than once per season, as well as sex. Capture (c) and recapture (p) probability were set to equal and were allowed to vary by previously described variables as well as environmental covariates such as rainfall, sea surface temperature (SST), Southern Oscillation Index (SOI), and Southern Annular Mode (SAM). *f*0 is a derived parameter and refers to the number of individuals not seen or ‘missed’ in closed population models

#### Locally weighted scatterplot smoothing regression

A locally weighted scatterplot smoothing regression (LOESS) works by fitting a separate weighted regression line to each abundance estimate in the scatterplot, where weighting emphasises nearby points and de-emphasises distant points, allowing for local trends to be captured whilst smoothing out variability in the data (Cleveland and Devlin, 1988, Cleveland and Loader, 1996). Incorporating weightings (variance) to the datapoints in the scatterplot allows LOESS to emphasise or de-emphasise certain datapoints based on their importance or reliability, thus, adjusting the influence of each datapoint on the local regression fitting process and minimising the effect of ‘noise’ (Cleveland and Devlin, 1988, Cleveland and Loader, 1996).

### 1.2 Validation of Model Assumptions and Goodness of Fit

**Table S1:** Validation of Pollock’s Robust Design capture-recapture model assumptions used for estimation of population demography of Indo-Pacific bottlenose dolphins *(Tursiops aduncus)* in the Adelaide Dolphin Sanctuary, South Australia (Parra et al., 2006)

| Assumption | Bias in estimates | Validation |
| --- | --- | --- |
| Marks are unique, permanent, and identified correctly | Upwards | - Use only good and excellent quality photographs to identify individuals. - Analysis restricted to individuals with long-lasting marks. - Regular sampling over 32 years permitted comprehensive monitoring of marked animals and any changes in their marks. |
| Equal capture and survival probabilities | Downwards | - Assess GOF using TEST 2 + TEST 3 to check for heterogeneity - Account for heterogeneity in modelling process using TSM approach. - High capture probabilities. - Knowledge of study area and study species (i.e., small area, small number of individuals) |
| No behavioural responses | Trap happy = downwards  Trap shy = upwards | - Trap response minimised with photo-identification techniques as animals are not subject to stress induced by capture, handling, or physical marking by the researcher. - Assess GOF using TEST2C.T |
| Instantaneous sampling for secondary periods | Upwards | - Secondary sampling occasions selected for analysis were relatively short (months) in comparison with the lifespan of the species (decades). |
| Independent captures | Upwards | - Assumption of independence is always violated for dolphins given that they occur in clusters. The violation of this assumption can lead to overdispersion of the data which can be adjusted using the variance inflation factor (*ĉ*). |

Goodness-of-fit (GOF) testing is used to assess departures from these model assumptions. There is no formal GOF testing for PRD, however, data can be collapsed to assess the GOF of the closed and open portions of the data. By collapsing the secondary periods we determined GOF across 112 primary sampling periods in program U-CARE (Choquet et al., 2005) using chi-squared tests for transience, trap-dependence, and capture probability (Lebreton et al., 1992). Estimation of the variance inflation factor (VIF) ($\hat{c}$) was used to quantify overdispersion using the chi-squared statistic from the Global Test (TEST 2 + TEST 3) divided by its degrees of freedom (Choquet et al., 2005, Haughey et al., 2020).

Closure is assumed within primary periods but not between them; to fulfil this assumption PRD relies on secondary periods being close together. In this study, secondary periods were defined by months, thus, closure was assumed given the short period of time where substantial demographic change within the population is unlikely to occur considering that bottlenose dolphins typically have a long lifespan (30+ years) and reproductive process. Additionally, the species showed a good degree of residency to the area as shown by multiple recaptures.

### 1.3 Environmental Data or Covariates

Climatic variation along the Australian coastline is dominated by the El Niño – Southern Oscillation (ENSO) phenomenon, a fluctuation in surface atmospheric pressure between the Australian-East Asian region and the eastern tropical Pacific resulting in extreme climatic and rainfall variation, often alternating between drought and flood dominated periods (McPhaden, 2002, Duignan et al., 2020). The Southern Annular Mode (SAM) is the dominant mode of atmospheric circulation variability and explains approximately 30% of climate variability in the Southern Hemisphere (Marshall, 2003). SAM refers to the non-seasonal north-south movement of strong westerly winds that blow almost continuously in the mid to high latitudes of Southern hemisphere (Marshall, 2003). Typically, a strengthening of SAM is associated with significant cooling over much of Australia, as well as increased rainfall in South-West Australia (Marshall, 2003). The Southern Oscillation Index (SOI) refers to the normalised difference in atmospheric pressure between Darwin and Tahiti and is a measure of the strength of ENSO phases (La Niña, Neutral, and El Niño) (Bureau of Meteorology, 2012, National Oceanic and Atmospheric Administration, 2024). Prolonged periods of positive SOI represent La Nina, and prolonged periods of negative SOI represent El Nino (Bureau of Meteorology, 2012).

**Table S2:** Sources of environmental covariate data used to estimate demographic parameters of Indo-Pacific bottlenose dolphins *(Tursiops aduncus)* in the Adelaide Dolphin Sanctuary, South Australia, using Pollock’s Robust Design capture-recapture model.

| **Environmental Variable** | **Data Source** |
| --- | --- |
| **Rainfall** | Monthly rainfall measures (mm) were obtained from the Australian Government Bureau of Meteorology: <http://www.bom.gov.au/climate/data/index.shtml>  Data was selected from the two weather stations that are closest to the Port River and that had data available for the full study period (1993 – 2020). The weather stations used were 023024 Adelaide (Seaton), SA (5.2 km away from Port River), and 023081 Bolivar Treatment Works, SA (11.6 km away from Port River). |
| **Sea surface temperature** | Monthly SST data was downloaded from the Australian Ocean Data Network (AODN) through the Integrated Marine Observing System (IMOS): <https://portal.aodn.org.au/search>  A subset of data was taken from the metadata source: IMOS – SRS – SST – L3S – Single Sensor – 1 month – day and nighttime – Australia using the spatial setting: Lat/Lon – 34.822, 138.436 to Lat/Lon – 34.721, 138.485 (the closest available data point to the study area), and temporal setting: 16^th^ March 1992 – 16^th^ Dec 2020. |
| **Southern Oscillation Index** | Monthly SOI estimates were obtained from National Oceanic and Atmospheric Administration (NOAA) National Centres for Environmental Information: <https://www.cpc.ncep.noaa.gov/data/indices/soi> |
| **Southern Annular Mode** | Monthly SAM data were extracted from the National Centre of Atmospheric Research Climate Data Guide (Marshall, 2003). |

### 1.4 Proportion of marked individuals

For seasonal abundance estimates, the proportion of marked individuals in the population was taken into account as follows (Wilson et al., 1999, Nicholson et al., 2012):

$$\hat{N}_{total}= \frac{\hat{N}_{m}}{\hat{\theta}}$$

Where $\hat{N}_{total}$ is the estimated abundance adjusted for the proportion of marked individuals, $\hat{N}_{m}$ is the estimated abundance of the marked population, and $\hat{\theta}$ is the estimated proportion of marked individuals. Standard errors of total population size were calculated as per Williams et al. (2002), where *n* is the total number of photographs from which $\hat{\theta}$ was derived:

$$SE\left( \hat{N}_{total} \right)=\sqrt{\hat{N}_{total}^{2}\left( \frac{SE{(\hat{N}_{m})}^{2}}{{\hat{N}_{m}}^{2}}+ \frac{1- \hat{\theta}}{n\hat{\theta}} \right)}$$

Lower and upper log-normal 95% confidence intervals were calculated as

$\hat{N}_{Lower total}= \frac{\hat{N}_{total}}{C}$, $\hat{N}_{Upper total}= \hat{N}_{total}\times C$ (Burnham, 1987), where:

$$C=\exp\left( 1.96 \sqrt{\ln\left( 1+ \left( \frac{SE\left( \hat{N}_{total} \right)}{\hat{N}_{total}} \right)^{2} \right)} \right)$$

### 1.5 Site fidelity

To calculate the standardised site fidelity index (SSFI) (Tschopp et al., 2018):

$$IT=\frac{IT individual 1+IT individual 2\ldots}{Total individuals}$$

and

$$It=\frac{It individual 1+It individual 2\ldots}{Total individuals}$$

Followed by:

$$\frac{2}{\frac{1}{IT}+\frac{1}{It}}$$

Where *IT* (permeance) represents the amount of time in the study area expressed as an average number of days between an individual’s first and last capture as a proportion of the total number of sampling days. *It* (periodicity) represents the average recurrence of an individual expressed as an inverse fraction of the number of days between an individual’s first and last capture as a proportion of the individual’s total number of captures minus one.

### 1.6 Residency

LIR is the probability that if an individual is identified within the study area at any one time, it is identified in the area some lag time later (Whitehead, 2001). Plots of LIR against time are used as an indication of the temporal use of the area by individual animals. If the population is closed, LIR should remain constant, if there is emigration and/or mortality the LIR will typically fall with time lag. When LIR drops after a certain time lag but continues to level off above zero, this indicates that some individuals may remain resident and/or other individuals re-immigrate into the study area (Whitehead, 2001).

## Results

### 2.1 Survey effort, photo-ID, and proportion of marked individuals

**Table S3:** Yearly and monthly boat survey effort (number of survey days) to study Indo-Pacific bottlenose dolphins *(Tursiops aduncus)* in the Adelaide Dolphin Sanctuary, South Australia, during 1993 – 2020.

|  | Jan | Feb | Mar | Apr | May | Jun | Jul | Aug | Sep | Oct | Nov | Dec | Total |
| --- | --- | --- | --- | --- | --- | --- | --- | --- | --- | --- | --- | --- | --- |
| 1993 | 6 | 2 | 8 | 11 | 2 | 5 | 1 | 2 | 5 | 4 | 3 | 3 | 52 |
| 1994 | 2 | 1 | 7 | 4 | 2 | 5 | 4 | 5 | 2 | 5 | 3 | 3 | 43 |
| 1995 | 4 | 0 | 4 | 5 | 2 | 1 | 1 | 4 | 4 | 5 | 3 | 5 | 38 |
| 1996 | 8 | 5 | 4 | 4 | 8 | 4 | 4 | 5 | 1 | 6 | 5 | 4 | 58 |
| 1997 | 5 | 3 | 1 | 9 | 5 | 5 | 4 | 4 | 8 | 3 | 3 | 5 | 55 |
| 1998 | 1 | 4 | 5 | 3 | 5 | 3 | 7 | 9 | 6 | 6 | 8 | 6 | 63 |
| 1999 | 8 | 6 | 11 | 10 | 7 | 6 | 6 | 8 | 4 | 6 | 10 | 4 | 86 |
| 2000 | 4 | 10 | 8 | 10 | 6 | 4 | 3 | 4 | 6 | 3 | 9 | 8 | 75 |
| 2001 | 11 | 3 | 7 | 6 | 9 | 5 | 7 | 2 | 6 | 5 | 10 | 10 | 81 |
| 2002 | 8 | 5 | 3 | 10 | 8 | 3 | 5 | 6 | 3 | 7 | 7 | 1 | 66 |
| 2003 | 11 | 7 | 6 | 9 | 9 | 5 | 10 | 2 | 3 | 5 | 11 | 9 | 87 |
| 2004 | 9 | 10 | 4 | 3 | 9 | 3 | 7 | 3 | 9 | 7 | 4 | 3 | 71 |
| 2005 | 5 | 4 | 7 | 3 | 5 | 3 | 4 | 3 | 6 | 5 | 3 | 1 | 49 |
| 2006 | 6 | 2 | 4 | 6 | 3 | 1 | 3 | 4 | 4 | 8 | 7 | 1 | 49 |
| 2007 | 7 | 6 | 3 | 3 | 3 | 2 | 4 | 6 | 3 | 8 | 3 | 4 | 52 |
| 2008 | 2 | 2 | 3 | 3 | 4 | 3 | 2 | 1 | 2 | 4 | 6 | 5 | 37 |
| 2009 | 4 | 3 | 4 | 2 | 0 | 4 | 2 | 3 | 3 | 4 | 4 | 2 | 35 |
| 2010 | 3 | 1 | 1 | 6 | 4 | 3 | 5 | 3 | 3 | 6 | 2 | 1 | 38 |
| 2011 | 8 | 2 | 4 | 1 | 4 | 6 | 3 | 5 | 1 | 3 | 2 | 4 | 43 |
| 2012 | 2 | 5 | 3 | 6 | 6 | 5 | 0 | 6 | 2 | 7 | 4 | 0 | 46 |
| 2013 | 6 | 4 | 2 | 4 | 0 | 4 | 3 | 4 | 4 | 5 | 5 | 1 | 42 |
| 2014 | 5 | 5 | 3 | 4 | 6 | 1 | 2 | 5 | 7 | 5 | 4 | 2 | 49 |
| 2015 | 5 | 2 | 3 | 0 | 4 | 3 | 1 | 0 | 2 | 0 | 0 | 0 | 20 |
| 2016 | 0 | 1 | 4 | 2 | 2 | 2 | 2 | 2 | 2 | 2 | 1 | 1 | 21 |
| 2017 | 1 | 3 | 1 | 1 | 0 | 2 | 1 | 5 | 0 | 1 | 2 | 1 | 18 |
| 2018 | 0 | 0 | 0 | 2 | 1 | 2 | 3 | 2 | 5 | 3 | 0 | 2 | 20 |
| 2019 | 1 | 0 | 3 | 2 | 5 | 5 | 5 | 4 | 5 | 5 | 3 | 3 | 41 |
| 2020 | 2 | 3 | 2 | 4 | 2 | 4 | 4 | 3 | 1 | 3 | 2 | 1 | 31 |
| Total | 134 | 99 | 115 | 133 | 121 | 99 | 103 | 110 | 105 | 131 | 124 | 90 | 1364 |

**Figure S*2*:** Cumulative discovery curves of newly identified Indo-Pacific bottlenose dolphins *(Tursiops aduncus)* in the Adelaide Dolphin Sanctuary, South Australia, between 1993 - 2020. Columns represent the number of survey days per month.

### 2.2 Goodness of fit

Rejection of TEST3.SR (z >0) suggested heterogeneity in capture probabilities, with newly marked individuals showing a consistently lower probability of being seen again than previously marked individuals (Choquet et al., 2005). This is a common occurrence in dolphin studies due to some exhibiting transiency, which was accounted for in the modelling process using the TSM approach. Highest levels of transience were detected within the ‘unknown sex’ group (z = 4.52, p = 0.00), with lower levels of transience for males (z = 0.92, p = 0.18) and females (z = 1.34, p = 0.09) (Table S4). TEST 2.CT showed significant levels of trap-dependence (i.e., trap-happy) (z <0) across all groups. When considering the small size of the study area and survey route (approx. 40 km), it is likely that the same individuals were frequently resighted rather than a ‘trap-happy’ behavioural response to initial capture, particularly because the ADS dolphins are already subject to a high volume of boat traffic concentrated in this small area.

**Table S4:** Results from goodness of fit tests run in program U-CARE for primary sampling periods (seasons) of surveys of Indo-Pacific bottlenose dolphin *(Tursiops aduncus)* in the Adelaide Dolphin Sanctuary, South Australia between 1993 - 2020.

| **Parameters** | **Test 3.SR** | | | **Test 2.CT** | | | **Global Test** | $\hat{\boldsymbol{c}}$ |
| --- | --- | --- | --- | --- | --- | --- | --- | --- |
|  | *Female* | *Male* | *Unknown* | *Female* | *Male* | *Unknown* |  |  |
| $\boldsymbol{\chi}^{\boldsymbol{2}}$ | 5.04 | 2.54 | 50.34 | 539.38 | 401.90 | 578.34 | 1985.23 | 2.46 |
| $\boldsymbol{\chi}^{\boldsymbol{2}}$ ***p-*value** | 0.75 | 0.47 | 0.21 | 0.00 | 0.00 | 0.00 | 0.00 |  |
| **z** | 1.34 | 0.92 | 4.52 | -19.59 | -17.81 | -19.80 |  |  |
| **z *p-*value** | 0.09 | 0.18 | 0.00 | 0.00 | 0.00 | 0.00 |  |  |
| **df** | 8 | 3 | 43 | 122 | 106 | 114 | 807 |  |

### 2.3 Abundance estimates

**Table S5:** Seasonal estimates of abundance (N) of Indo-Pacific bottlenose dolphins *(Tursiops aduncus)* in the Adelaide Dolphin Sanctuary, South Australia, from 1993 – 2020. Nm: estimated number of marked animals in the population; Ntotal: estimate of total population size after correcting for proportion of identified individuals. CIs that are “na” represent intervals where the standard error was zero.

| Year | Season | *Nm* | 95% CI | *Ntotal* | 95% CI |
| --- | --- | --- | --- | --- | --- |
| 1993 | **SUMMER** | 35.73 | 35.69 – 35.76 | 59.99 | na |
|  | **AUTUMN** | 25.00 | na | 41.98 | na |
|  | **WINTER** | 14.00 | na | 23.51 | na |
|  | **SPRING** | 23.00 | na | 38.62 | na |
| 1994 | **SUMMER** | 19.68 | 18.21 – 31.25 | 33.04 | 25.17 – 40.92 |
|  | **AUTUMN** | 25.62 | 25.04 – 35.34 | 43.02 | 37.71 – 48.33 |
|  | **WINTER** | 22.00 | na | 36.94 | na |
|  | **SPRING** | 26.68 | 25.26 – 35.82 | 44.79 | 38.12 – 51.46 |
| 1995 | **SUMMER** | 26.02 | 24.37 – 34.90 | 43.69 | 36.75 – 50.62 |
|  | **AUTUMN** | 29.46 | 28.20 – 38.63 | 49.47 | 43.07 – 55.87 |
|  | **WINTER** | 22.87 | 20.65 – 32.71 | 38.41 | 30.08 – 46.73 |
|  | **SPRING** | 23.00 | 23.00 – 23.00 | 38.62 | na |
| 1996 | **SUMMER** | 36.00 | na | 60.45 | na |
|  | **AUTUMN** | 28.00 | 28.00 – 28.00 | 47.02 | na |
|  | **WINTER** | 25.58 | 25.03 – 35.55 | 42.95 | 37.60 – 48.30 |
|  | **SPRING** | 20.00 | 20.00 – 20.00 | 33.58 | na |
| 1997 | **SUMMER** | 24.50 | 24.02 – 34.58 | 41.13 | 35.88 – 46.38 |
|  | **AUTUMN** | 25.00 | 25.00 – 25.00 | 41.98 | na |
|  | **WINTER** | 18.00 | na | 30.23 | na |
|  | **SPRING** | 24.00 | na | 40.30 | na |
| 1998 | **SUMMER** | 22.32 | 22.01 – 33.08 | 37.48 | 32.20 – 42.76 |
|  | **AUTUMN** | 22.18 | 22.00 – 32.30 | 37.24 | 32.30 – 42.18 |
|  | **WINTER** | 23.00 | 23.00 – 23.00 | 38.62 | na |
|  | **SPRING** | 28.00 | na | 47.02 | na |
| 1999 | **SUMMER** | 37.00 | na | 62.13 | na |
|  | **AUTUMN** | 33.00 | 33.00 – 33.00 | 55.41 | - |
|  | **WINTER** | 25.00 | na | 41.98 | na |
|  | **SPRING** | 32.00 | 32.00 – 32.00 | 53.73 | na |
| 2000 | **SUMMER** | 43.00 | na | 72.20 | na |
|  | **AUTUMN** | 31.00 | 31.00 – 31.00 | 52.05 | na |
|  | **WINTER** | 24.36 | 23.18 – 33.47 | 40.91 | 34.66 – 47.15 |
|  | **SPRING** | 46.00 | 46.00 – 46.02 | 77.24 | na |
| 2001 | **SUMMER** | 59.00 | na | 99.07 | na |
|  | **AUTUMN** | 52.00 | 52.00 – 52.00 | 87.32 | na |
|  | **WINTER** | 40.13 | 39.13 – 49.02 | 67.39 | 61.62 – 73.16 |
|  | **SPRING** | 42.00 | na | 70.53 | na |
| 2002 | **SUMMER** | 49.63 | 48.26 – 58.00 | 83.33 | 77.18 – 89.49 |
|  | **AUTUMN** | 49.00 | 49.00 – 49.00 | 82.28 | na |
|  | **WINTER** | 34.48 | 34.02 – 44.66 | 57.90 | 52.66 – 63.14 |
|  | **SPRING** | 35.00 | na | 58.77 | na |
| 2003 | **SUMMER** | 65.00 | na | 109.15 | na |
|  | **AUTUMN** | 53.00 | 53.00 – 53.00 | 89.00 | na |
|  | **WINTER** | 46.08 | 46.00 – 54.58 | 77.37 | 72.89 – 81.85 |
|  | **SPRING** | 53.00 | 53.00 – 53.00 | 89.00 | na |
| 2004 | **SUMMER** | 75.00 | 75.00 – 75.00 | 125.94 | na |
|  | **AUTUMN** | 56.08 | 55.12 – 64.84 | 94.18 | 88.60 – 99.75 |
|  | **WINTER** | 48.75 | 46.69 – 56.94 | 81.85 | 74.67 – 89.03 |
|  | **SPRING** | 69.00 | na | 115.86 | na |
| 2005 | **SUMMER** | 83.64 | 78.73 – 93.64 | 140.44 | 128.66 – 152.22 |
|  | **AUTUMN** | 72.02 | 69.82 – 80.10 | 120.94 | 113.70 – 128.18 |
|  | **WINTER** | 60.12 | 56.06 – 69.53 | 100.94 | 90.46 – 111.43 |
|  | **SPRING** | 46.08 | 44.42 – 54.33 | 77.38 | 70.77 – 83.99 |
| 2006 | **SUMMER** | 62.49 | 58.30 – 72.01 | 104.93 | 94.23 – 115.63 |
|  | **AUTUMN** | 59.94 | 57.27 – 68.27 | 100.66 | 92.56 – 108.75 |
|  | **WINTER** | 33.57 | 30.53 – 42.64 | 56.37 | 47.31 – 65.44 |
|  | **SPRING** | 53.00 | 53.00 – 54.69 | 89.00 | 86.04 – 91.96 |
| 2007 | **SUMMER** | 60.15 | 59.14 – 68.82 | 101.01 | 95.39 – 106.63 |
|  | **AUTUMN** | 49.67 | 45.76 – 59.13 | 83.41 | 73.05 – 93.78 |
|  | **WINTER** | 58.54 | 55.58 – 67.03 | 98.29 | 89.73 – 106.86 |
|  | **SPRING** | 46.65 | 45.27 – 55.05 | 78.33 | 72.13 – 84.54 |
| 2008 | **SUMMER** | 41.10 | 37.85 – 50.08 | 69.02 | 59.75 – 78.29 |
|  | **AUTUMN** | 48.60 | 45.15 – 57.62 | 81.61 | 72.08 – 91.15 |
|  | **WINTER** | 54.36 | 48.72 – 65.57 | 91.28 | 77.82 – 104.74 |
|  | **SPRING** | 79.42 | 75.68 – 88.38 | 133.37 | 123.60 – 143.13 |
| 2009 | **SUMMER** | 40.10 | 36.84 – 49.14 | 67.34 | 58.02 – 76.66 |
|  | **AUTUMN** | 44.37 | 39.75 – 54.70 | 74.51 | 62.76 – 86.26 |
|  | **WINTER** | 56.99 | 52.60 – 66.73 | 95.69 | 84.60 – 106.78 |
|  | **SPRING** | 81.85 | 77.21 – 91.58 | 137.44 | 126.14 – 148.73 |
| 2010 | **SUMMER** | 50.66 | 44.86 – 62.19 | 85.06 | 71.21 – 98.91 |
|  | **AUTUMN** | 54.78 | 51.71 – 63.39 | 91.99 | 83.19 – 100.78 |
|  | **WINTER** | 53.27 | 49.97 – 62.05 | 89.44 | 80.26 – 98.62 |
|  | **SPRING** | 50.50 | 47.55 – 59.08 | 84.79 | 76.16 – 93.43 |
| 2011 | **SUMMER** | 79.93 | 77.27 – 88.12 | 134.21 | 126.30 – 142.12 |
|  | **AUTUMN** | 70.76 | 70.39 – 71.16 | 118.83 | na |
|  | **WINTER** | 66.66 | 64.13 – 74.87 | 111.94 | 104.13 – 119.74 |
|  | **SPRING** | 62.15 | 55.97 – 73.84 | 104.37 | 90.01 – 118.72 |
| 2012 | **SUMMER** | 58.75 | 53.72 – 69.16 | 98.66 | 86.41 – 110.91 |
|  | **AUTUMN** | 62.64 | 60.65 – 70.72 | 105.18 | 98.21 – 112.15 |
|  | **WINTER** | 58.78 | 55.72 – 67.34 | 98.71 | 89.96 – 107.46 |
|  | **SPRING** | 54.10 | 51.85 – 62.29 | 90.84 | 83.40 – 98.28 |
| 2013 | **SUMMER** | 70.55 | 66.75 – 79.58 | 118.47 | 108.56 – 128.37 |
|  | **AUTUMN** | 52.21 | 46.97 – 63.01 | 87.68 | 74.92 – 100.43 |
|  | **WINTER** | 52.28 | 48.98 – 61.08 | 87.78 | 78.58 – 96.99 |
|  | **SPRING** | 46.22 | 44.47 – 54.46 | 77.62 | 70.87 – 84.36 |
| 2014 | **SUMMER** | 60.89 | 57.77 – 69.46 | 102.24 | 93.43 – 111.05 |
|  | **AUTUMN** | 64.37 | 61.50 – 72.77 | 108.09 | 99.71 – 116.48 |
|  | **WINTER** | 47.98 | 43.95 – 57.54 | 80.57 | 70.00 – 91.14 |
|  | **SPRING** | 78.87 | 76.75 – 86.89 | 132.43 | 125.38 – 139.48 |
| 2015 | **SUMMER** | 76.73 | 70.76 – 87.97 | 128.84 | 115.05 – 142.63 |
|  | **AUTUMN** | 71.48 | 65.57 – 82.69 | 120.02 | 106.31 – 133.74 |
|  | **WINTER** | 43.85 | 38.26 – 55.40 | 73.64 | 60.00 – 87.28 |
|  | **SPRING** | 22.22 | 18.25 – 33.19 | 37.31 | 25.94 – 48.67 |
| 2016 | **SUMMER** | 33.28 | 27.72 – 45.40 | 55.88 | 41.91 – 69.85 |
|  | **AUTUMN** | 72.40 | 66.84 – 83.28 | 121.58 | 108.46 – 134.69 |
|  | **WINTER** | 52.81 | 47.31 – 63.99 | 88.67 | 75.39 – 101.96 |
|  | **SPRING** | 45.57 | 40.11 – 56.86 | 76.52 | 63.20 – 89.83 |
| 2017 | **SUMMER** | 70.49 | 63.28 – 83.29 | 118.36 | 102.17 – 134.55 |
|  | **AUTUMN** | 48.30 | 41.12 – 61.82 | 81.11 | 64.43 – 97.78 |
|  | **WINTER** | 45.38 | 41.57 – 54.80 | 76.20 | 65.98 – 86.41 |
|  | **SPRING** | 31.76 | 26.83 – 43.06 | 53.34 | 40.60 – 66.07 |
| 2018 | **SUMMER** | 38.70 | 32.66 – 51.20 | 64.98 | 50.22 – 79.75 |
|  | **AUTUMN** | 43.31 | 37.16 – 55.61 | 72.73 | 57.98 – 87.47 |
|  | **WINTER** | 38.78 | 34.77 – 48.59 | 65.11 | 54.40 – 75.82 |
|  | **SPRING** | 30.77 | 28.13 – 39.58 | 51.66 | 43.28 – 60.04 |
| 2019 | **SUMMER** | 53.05 | 46.75 – 65.25 | 89.09 | 74.25 – 103.93 |
|  | **AUTUMN** | 44.77 | 41.68 – 53.57 | 75.18 | 66.22 – 84.14 |
|  | **WINTER** | 40.81 | 39.32 – 49.18 | 68.53 | 62.13 – 74.92 |
|  | **SPRING** | 44.69 | 42.66 – 52.93 | 75.04 | 67.86 – 82.21 |
| 2020 | **SUMMER** | 50.19 | 44.92 – 61.12 | 84.28 | 71.40 – 97.15 |
|  | **AUTUMN** | 36.38 | 32.98 – 45.61 | 61.09 | 51.48 – 70.71 |
|  | **WINTER** | 34.93 | 32.75 – 43.41 | 58.65 | 51.11 – 66.19 |
|  | **SPRING** | 34.74 | 30.72 – 44.73 | 58.34 | 47.50 – 69.18 |

### 2.4 Apparent survival and temporary emigration

**Table *S*6:** Estimates of Markovian temporary emigration (γ”, γ’) from best fitting Pollock's Robust Design mark-recapture model for Indo-Pacific bottlenose dolphins (*Tursiops aduncus)* in the Adelaide Dolphin Sanctuary, South Australia, between 1993 - 2020. γ” is the probability of being a temporary emigrant if an animal was present in the previous primary period (season), and γ’ is the probability of being a temporary emigrant if an animal was absent in the previous primary period (i.e., remaining out of the study area). Emigration for the interval between the first primary period (1993 Summer – Autumn) cannot be estimated since it is a first sampling occasion with no previous sampling occasion from which to derive and estimate. γ’ estimates begin one primary period later given there are no animals to be considered in 1993 Autumn - Winter that were also outside of the study area in 1993 Summer - Autumn (i.e., they had not been captured yet).

| Year | Seasons | γ" | 95% CI | γ' | 95%CI |
| --- | --- | --- | --- | --- | --- |
| 1993 | **Autumn - Winter** | 0.36 | 0.20 – 0.55 | - | - |
|  | **Winter - Spring** | 0.34 | 0.13 – 0.65 | 0.91 | 0.46 – 0.99 |
|  | **Spring - Summer** | 0.07 | 0.00 – 0.63 | 0.63 | 0.29 – 0.88 |
| 1994 | **Summer - Autumn** | 0.25 | 0.08 – 0.54 | 0.81 | 0.34 – 0.97 |
|  | **Autumn - Winter** | 0.19 | 0.05 – 0.50 | 0.64 | 0.33 – 0.87 |
|  | **Winter - Spring** | 0.36 | 0.18 – 0.59 | 0.82 | 0.45 – 0.96 |
|  | **Spring - Summer** | 0.21 | 0.07 – 0.48 | 0.55 | 0.30 – 0.77 |
| 1995 | **Summer - Autumn** | 0.16 | 0.02 – 0.65 | 0.87 | 0.43 – 0.98 |
|  | **Autumn - Winter** | 0.06 | 0.01 – 0.42 | 0.67 | 0.29 – 0.91 |
|  | **Winter - Spring** | 0.13 | 0.03 – 0.41 | 1.00 | 0.00 – 1.00 |
|  | **Spring - Summer** | 0.00 | 0.00 – 1.00 | 0.86 | 0.41 – 0.98 |
| 1996 | **Summer - Autumn** | 0.06 | 0.01 – 0.28 | 0.66 | 0.29 – 0.90 |
|  | **Autumn - Winter** | 0.30 | 0.16 – 0.49 | 0.94 | 0.45 – 1.00 |
|  | **Winter - Spring** | 0.17 | 0.07 – 0.39 | 0.82 | 0.52 – 0.95 |
|  | **Spring - Summer** | 0.13 | 0.03 – 0.39 | 0.97 | 0.39 – 1.00 |
| 1997 | **Summer - Autumn** | 0.09 | 0.02 – 0.38 | 0.90 | 0.61 – 0.98 |
|  | **Autumn - Winter** | 0.19 | 0.07 – 0.42 | 0.79 | 0.56 – 0.91 |
|  | **Winter - Spring** | 0.23 | 0.10 – 0.46 | 1.00 | 0.00 – 1.00 |
|  | **Spring - Summer** | 0.00 | 0.00 – 1.00 | 0.79 | 0.59 – 0.91 |
| 1998 | **Summer - Autumn** | 0.26 | 0.10 – 0.52 | 0.83 | 0.59 – 0.94 |
|  | **Autumn - Winter** | 0.20 | 0.06 – 0.50 | 0.80 | 0.54 – 0.93 |
|  | **Winter - Spring** | 0.23 | 0.10 – 0.44 | 0.93 | 0.50 – 0.99 |
|  | **Spring - Summer** | 0.34 | 0.17 – 0.58 | 0.59 | 0.41 - 0.76 |
| 1999 | **Summer - Autumn** | 0.22 | 0.09 – 0.45 | 0.58 | 0.38 – 0.76 |
|  | **Autumn - Winter** | 0.22 | 0.11 – 0.39 | 0.84 | 0.55 – 0.96 |
|  | **Winter - Spring** | 0.29 | 0.16 – 0.48 | 0.89 | 0.68 – 0.97 |
|  | **Spring - Summer** | 0.03 | 0.00 – 0.26 | 0.77 | 0.58 – 0.89 |
| 2000 | **Summer - Autumn** | 0.10 | 0.03 – 0.28 | 0.56 | 0.36 – 0.36 |
|  | **Autumn - Winter** | 0.30 | 0.18 – 0.45 | 1.00 | 0.00 – 1.00 |
|  | **Winter - Spring** | 0.19 | 0.06 – 0.44 | 0.93 | 0.72 – 0.99 |
|  | **Spring - Summer** | 0.02 | 0.00 – 0.95 | 0.44 | 0.27 – 0.63 |
| 2001 | **Summer - Autumn** | 0.13 | 0.05 – 0.27 | 0.52 | 0.25 – 0.78 |
|  | **Autumn - Winter** | 0.22 | 0.12 – 0.35 | 0.65 | 0.35 – 0.86 |
|  | **Winter - Spring** | 0.29 | 0.17 – 0.45 | 0.72 | 0.47 – 0.88 |
|  | **Spring - Summer** | 0.24 | 0.13 – 0.41 | 0.73 | 0.51 – 0.87 |
| 2002 | **Summer - Autumn** | 0.20 | 0.10 – 0.37 | 0.58 | 0.39 – 0.75 |
|  | **Autumn - Winter** | 0.14 | 0.06 – 0.28 | 0.80 | 0.56 – 0.93 |
|  | **Winter - Spring** | 0.31 | 0.18 – 0.47 | 0.95 | 0.72 – 0.99 |
|  | **Spring - Summer** | 0.21 | 0.09 – 0.40 | 0.88 | 0.69 – 0.96 |
| 2003 | **Summer - Autumn** | 0.11 | 0.04 – 0.28 | 0.48 | 0.33 – 0.63 |
|  | **Autumn - Winter** | 0.24 | 0.14 – 0.36 | 0.78 | 0.57 – 0.90 |
|  | **Winter - Spring** | 0.22 | 0.12 – 0.37 | 0.83 | 0.65 – 0.93 |
|  | **Spring - Summer** | 0.03 | 0.00 – 0.29 | 0.73 | 0.56 – 0.86 |
| 2004 | **Summer - Autumn** | 0.11 | 0.04 – 0.24 | 0.39 | 0.22 – 0.58 |
|  | **Autumn - Winter** | 0.28 | 0.18 – 0.39 | 0.81 | 0.49 – 0.95 |
|  | **Winter - Spring** | 0.00 | 0.00 – 1.00 | 0.98 | 0.63 – 1.00 |
|  | **Spring - Summer** | 0.00 | 0.00 – 0.00 | 0.62 | 0.44 – 0.78 |
| 2005 | **Summer - Autumn** | 0.00 | 0.00 – 0.00 | 0.44 | 0.23 – 0.67 |
|  | **Autumn - Winter** | 0.17 | 0.10 – 0.29 | 1.00 | 0.00 – 1.00 |
|  | **Winter - Spring** | 0.19 | 0.10 – 0.32 | 1.00 | 0.00 – 1.00 |
|  | **Spring - Summer** | 0.20 | 0.10 – 0.37 | 0.93 | 0.72 – 0.99 |
| 2006 | **Summer - Autumn** | 0.02 | 0.00 – 0.78 | 0.63 | 0.44 – 0.78 |
|  | **Autumn - Winter** | 0.21 | 0.12 – 0.35 | 0.75 | 0.51 – 0.90 |
|  | **Winter - Spring** | 0.39 | 0.24 – 0.57 | 0.95 | 0.70 – 0.99 |
|  | **Spring - Summer** | 0.03 | 0.00 – 0.40 | 0.67 | 0.51 – 0.80 |
| 2007 | **Summer - Autumn** | 0.09 | 0.03 – 0.28 | 0.61 | 0.44 – 0.76 |
|  | **Autumn - Winter** | 0.26 | 0.14 – 0.42 | 0.88 | 0.52 – 0.98 |
|  | **Winter - Spring** | 0.13 | 0.05 – 0.28 | 0.70 | 0.50 – 0.84 |
|  | **Spring - Summer** | 0.22 | 0.12 – 0.37 | 0.89 | 0.66 – 0.97 |
| 2008 | **Summer - Autumn** | 0.17 | 0.06 – 0.39 | 0.96 | 0.68 – 1.00 |
|  | **Autumn - Winter** | 0.17 | 0.07 – 0.36 | 0.75 | 0.56 – 0.87 |
|  | **Winter - Spring** | 0.05 | 0.00 – 0.45 | 0.86 | 0.64 – 0.95 |
|  | **Spring - Summer** | 0.03 | 0.00 – 0.47 | 0.46 | 0.28 – 0.64 |
| 2009 | **Summer - Autumn** | 0.52 | 0.38 – 0.65 | 0.93 | 0.42 – 1.00 |
|  | **Autumn - Winter** | 0.10 | 0.02 – 0.35 | 0.88 | 0.66 – 0.97 |
|  | **Winter - Spring** | 0.03 | 0.00 – 0.38 | 0.80 | 0.63 – 0.90 |
|  | **Spring - Summer** | 0.00 | 0.00 – 0.00 | 0.44 | 0.28 – 0.62 |
| 2010 | **Summer - Autumn** | 0.32 | 0.21 – 0.46 | 0.92 | 0.38 – 1.00 |
|  | **Autumn - Winter** | 0.01 | 0.00 – 0.93 | 0.96 | 0.23 – 1.00 |
|  | **Winter - Spring** | 0.21 | 0.11 – 0.37 | 0.76 | 0.58 – 0.88 |
|  | **Spring - Summer** | 0.14 | 0.05 – 0.33 | 0.91 | 0.68 – 0.98 |
| 2011 | **Summer - Autumn** | 0.15 | 0.06 – 0.32 | 0.43 | 0.28 – 0.60 |
|  | **Autumn - Winter** | 0.27 | 0.16 – 0.42 | 0.49 | 0.27 – 0.71 |
|  | **Winter - Spring** | 0.15 | 0.07 – 0.29 | 0.83 | 0.50 – 0.96 |
|  | **Spring - Summer** | 0.19 | 0.09 – 0.36 | 0.72 | 0.51 – 0.86 |
| 2012 | **Summer - Autumn** | 0.05 | 0.00 – 0.52 | 0.94 | 0.47 – 1.00 |
|  | **Autumn - Winter** | 0.18 | 0.07 – 0.39 | 0.62 | 0.43 – 0.78 |
|  | **Winter - Spring** | 0.15 | 0.08 – 0.29 | 0.96 | 0.02 – 1.00 |
|  | **Spring - Summer** | 0.18 | 0.08 – 0.37 | 0.76 | 0.59 – 0.87 |
| 2013 | **Summer - Autumn** | 0.09 | 0.02 – 0.27 | 0.65 | 0.45 – 0.80 |
|  | **Autumn - Winter** | 0.28 | 0.16 – 0.43 | 0.92 | 0.61 – 0.99 |
|  | **Winter - Spring** | 0.16 | 0.07 – 0.33 | 0.85 | 0.64 – 0.95 |
|  | **Spring - Summer** | 0.11 | 0.03 – 0.35 | 0.91 | 0.74 – 0.97 |
| 2014 | **Summer - Autumn** | 0.09 | 0.02 – 0.27 | 0.59 | 0.43 – 0.74 |
|  | **Autumn - Winter** | 0.08 | 0.02 – 0.22 | 0.79 | 0.56 – 0.92 |
|  | **Winter - Spring** | 0.24 | 0.12 – 0.42 | 1.00 | 0.00 – 1.00 |
|  | **Spring - Summer** | 0.00 | 0.00 – 1.00 | 0.40 | 0.25 – 0.57 |
| 2015 | **Summer - Autumn** | 0.07 | 0.02 – 0.23 | 0.49 | 0.22 – 0.76 |
|  | **Autumn - Winter** | 0.09 | 0.03 – 0.28 | 0.95 | 0.01 – 1.00 |
|  | **Winter - Spring** | 0.38 | 0.24 – 0.54 | 1.00 | 0.00 – 1.00 |
|  | **Spring - Summer** | 0.33 | 0.14 – 0.60 | 1.00 | 0.00 – 1.00 |
| 2016 | **Summer - Autumn** | 0.00 | 0.00 – 0.00 | 0.84 | 0.61 – 0.95 |
|  | **Autumn - Winter** | 0.03 | 0.00 – 0.80 | 0.36 | 0.19 – 0.58 |
|  | **Winter - Spring** | 0.27 | 0.15 – 0.44 | 0.68 | 0.25 – 0.93 |
|  | **Spring - Summer** | 0.08 | 0.01 – 0.57 | 0.99 | 0.00 – 1.00 |
| 2017 | **Summer - Autumn** | 0.08 | 0.01 – 0.40 | 0.00 | 0.00 – 1.00 |
|  | **Autumn - Winter** | 0.44 | 0.32 – 0.57 | 0.00 | 0.00 – 1.00 |
|  | **Winter - Spring** | 0.06 | 0.01 – 0.28 | 1.00 | 1.00 – 1.00 |
|  | **Spring - Summer** | 0.06 | 0.00 – 0.51 | 1.00 | 0.00 – 1.00 |
| 2018 | **Summer - Autumn** | 0.00 | 0.00 – 1.00 | 0.72 | 0.52 – 0.86 |
|  | **Autumn - Winter** | 0.05 | 0.00 – 0.44 | 1.00 | 1.00 – 1.00 |
|  | **Winter - Spring** | 0.23 | 0.11 – 0.43 | 1.00 | 1.00 – 1.00 |
|  | **Spring - Summer** | 0.07 | 0.01 – 0.35 | 1.00 | 0.00 – 1.00 |
| 2019 | **Summer - Autumn** | 0.00 | 0.00 – 1.00 | 0.75 | 0.52 – 0.89 |
|  | **Autumn - Winter** | 0.16 | 0.06 – 0.34 | 0.78 | 0.51 – 0.93 |
|  | **Winter - Spring** | 0.12 | 0.04 – 0.30 | 0.93 | 0.63 – 0.99 |
|  | **Spring - Summer** | 0.09 | 0.02 – 0.29 | 0.72 | 0.51 – 0.86 |
| 2020 | **Summer - Autumn** | 0.10 | 0.03 – 0.32 | 0.77 | 0.52 – 0.91 |
|  | **Autumn - Winter** | 0.23 | 0.11 – 0.43 | 1.00 | 1.00 – 1.00 |
|  | **Winter - Spring** | 0.12 | 0.03 – 0.38 | 0.90 | 0.68 – 0.97 |
|  | **Spring - Summer** | 0.19 | 0.05 – 0.51 | 0.75 | 0.52 – 0.89 |

## References

ADMINISTRATION, N. O. A. A. 2024. *El Nino/Southern Oscillation (ENSO)* [Online]. Available: <https://www.ncei.noaa.gov/access/monitoring/enso/soi> [Accessed].

BURNHAM, K. P. 1987. *Design and analysis methods for fish survival experiments based on release-recapture*, American Fisheries Society.

CHOQUET, R., et al. 2005. U-CARE 2.2 user’s manual. *CEFE, Montpellier, France,* 53.

CLEVELAND, W. S. & DEVLIN, S. J. 1988. Locally weighted regression: an approach to regression analysis by local fitting. *Journal of the American statistical association,* 83**,** 596-610.

CLEVELAND, W. S. & LOADER, C. Smoothing by local regression: Principles and methods. Statistical Theory and Computational Aspects of Smoothing: Proceedings of the COMPSTAT’94 Satellite Meeting held in Semmering, Austria, 27–28 August 1994, 1996. Springer, 10-49.

COOCH, E. & WHITE, G. 2014. *Program MARK: A Gentle Introduction*, Colorado State University.

HAUGHEY, R., et al. 2020. Photographic Capture-Recapture Analysis Reveals a Large Population of Indo-Pacific Bottlenose Dolphins (Tursiops aduncus) With Low Site Fidelity off the North West Cape, Western Australia. *Frontiers in Marine Science,* 6.

KENDALL, W. L., et al. 1997. Estimating temporary emigration using capture–recapture data with Pollock’s robust design. *Ecology,* 78**,** 563-578.

LEBRETON, J.-D., et al. 1992. Modeling survival and testing biological hypotheses using marked animals: a unified approach with case studies. *Ecological monographs,* 62**,** 67-118.

MARSHALL, G. J. 2003. Trends in the Southern Annular Mode from observations and reanalyses. *Journal of Climate,* 16**,** 4134-4143.

METEOROLOGY, B. O. 2012. Record-breaking La Nina events: An analysis of the La Nina life cycle and the impacts and significance of the 2010-11 and 2011-2012 La Nina events in Australia. Melbourne: Australian Government

NICHOLSON, K., et al. 2012. Abundance, survival and temporary emigration of bottlenose dolphins (Tursiops sp.) off Useless Loop in the western gulf of Shark Bay, Western Australia. *Marine and Freshwater Research,* 63**,** 1059-1068.

PARRA, G. J., et al. 2006. Population sizes, site fidelity and residence patterns of Australian snubfin and Indo-Pacific humpback dolphins: Implications for conservation. *Biological Conservation,* 129**,** 167-180.

POLLOCK, K. H. 2000. Capture-recapture models. *Journal of the American Statistical Association,* 95**,** 293-296.

POLLOCK, K. H., et al. 1990. Statistical inference for capture-recapture experiments. *Wildlife monographs***,** 3-97.

SANDERCOCK, B. K. 2020. Mark-recapture models for estimation of demographic parameters. *Population ecology in practice***,** 157-190.

TSCHOPP, A., et al. 2018. Development of a site fidelity index based on population capture-recapture data. *PeerJ,* 6**,** e4782.

WHITEHEAD, H. 2001. Analysis of animal movement using opportunistic individual identifications: application to sperm whales. *Ecology,* 82**,** 1417-1432.

WILLIAMS, B. K., et al. 2002. *Analysis and management of animal populations*, Academic press.

WILSON, B., et al. 1999. Estimating size and assessing trends in a coastal bottlenose dolphin population. *Ecological applications,* 9**,** 288-300.
